# Plasmonically enhanced mid-IR light source based on tunable spectrally and directionally selective thermal emission from nanopatterned graphene

**DOI:** 10.1038/s41598-020-73582-3

**Published:** 2020-10-16

**Authors:** Muhammad Waqas Shabbir, Michael N. Leuenberger

**Affiliations:** 1 NanoScience Technology Center and Department of Physics, University of Central Florida, Orlando, FL 32826 USA; 2 College of Optics and Photonics, University of Central Florida, Orlando, FL 32826 USA

**Keywords:** Optics and photonics, Physics

## Abstract

We present a proof of concept for a spectrally selective thermal mid-IR source based on nanopatterned graphene (NPG) with a typical mobility of CVD-grown graphene (up to 3000 $\hbox {cm}^2\,\hbox {V}^{-1}\,\hbox {s}^{-1}$), ensuring scalability to large areas. For that, we solve the electrostatic problem of a conducting hyperboloid with an elliptical wormhole in the presence of an *in-plane* electric field. The localized surface plasmons (LSPs) on the NPG sheet, partially hybridized with graphene phonons and surface phonons of the neighboring materials, allow for the control and tuning of the thermal emission spectrum in the wavelength regime from $\lambda =3$ to 12 $\upmu$m by adjusting the size of and distance between the circular holes in a hexagonal or square lattice structure. Most importantly, the LSPs along with an optical cavity increase the emittance of graphene from about 2.3% for pristine graphene to 80% for NPG, thereby outperforming state-of-the-art pristine graphene light sources operating in the near-infrared by at least a factor of 100. According to our COMSOL calculations, a maximum emission power per area of $11\times 10^3$ W/$\hbox {m}^2$ at $T=2000$ K for a bias voltage of $V=23$ V is achieved by controlling the temperature of the hot electrons through the Joule heating. By generalizing Planck’s theory to any grey body and deriving the completely general nonlocal fluctuation-dissipation theorem with nonlocal response of surface plasmons in the random phase approximation, we show that the coherence length of the graphene plasmons and the thermally emitted photons can be as large as 13 $\upmu$m and 150 $\upmu$m, respectively, providing the opportunity to create phased arrays made of nanoantennas represented by the holes in NPG. The spatial phase variation of the coherence allows for beamsteering of the thermal emission in the range between $12^\circ$ and $80^\circ$ by tuning the Fermi energy between $E_F=1.0$ eV and $E_F=0.25$ eV through the gate voltage. Our analysis of the nonlocal hydrodynamic response leads to the conjecture that the diffusion length and viscosity in graphene are frequency-dependent. Using finite-difference time domain calculations, coupled mode theory, and RPA, we develop the model of a mid-IR light source based on NPG, which will pave the way to graphene-based optical mid-IR communication, mid-IR color displays, mid-IR spectroscopy, and virus detection.

## Introduction

An object that is kept in equilibrium at a given temperature $T>0$ K emits electromagnetic (EM) radiation because the charge carriers on the atomic and molecular scale oscillate due to their heat energy^1^. Planck’s law describes quantitatively the energy density $u(\omega )$ of the EM radiation per unit frequency $\omega$ for black-body radiation, which is $u_{BB}(\omega )d\omega =\frac{\omega ^2}{\pi ^2c^3}\Theta (\omega )d\omega$, where *c* is the speed of light in vacuum, $\hbar$ is the Planck constant, and $k_B$ is the Boltzmann constant. $\Theta (\omega ,T)=\hbar \omega /[\exp (\hbar \omega /k_BT)-1]$ is the thermal energy of a photon mode. Consequently, the energy emitted per unit surface area and per unit frequency, also called spectral radiance, of a black body into three-dimensional (3D) space is given by

1 $$\begin{aligned} I_{BB}(\omega )d\omega =\frac{1}{4\pi }cu(\omega )=\frac{\omega ^2}{4\pi ^3c^2} \Theta (\omega )d\omega . \end{aligned}$$

The total energy density *u* can then be obtained by integrating over all frequencies and angles over the half-sphere, leading to the Stefan-Boltzmann law for the energy density of black-body radiation,

2 $$\begin{aligned} u_{BB}=\left( \frac{8\pi ^5k_B^4}{15c^3h^3}\right) T^4=a_{BB}T^4, \end{aligned}$$

with $a_{BB}=7.566\times 10^{-16}$ $\hbox {Jm}^{-3}\,\hbox {K}^{-4}$. The total power emitted per unit surface area *P*/*A* of a black-body is

3 $$\begin{aligned} I_{BB}& = {} \frac{P}{A}=\int \limits _0^\infty I_{BB}(\omega )d\omega \int \limits _0^{2\pi }d\varphi \int \limits _0^{\pi /2} \cos \theta \sin \theta d\theta \nonumber \\& = {} \pi \int \limits _0^\infty I_{BB}(\omega )d\omega = \frac{1}{4\pi }uc \nonumber \\& = {} \frac{a_{BB}c}{4\pi }T^4=b_{BB} T^4=\left( \frac{\pi ^2k_B^4}{60c^2\hbar ^3}\right) T^4, \end{aligned}$$

where $b_{BB}=5.67\times 10^{-8}$ $\hbox {Wm}^{-2}\,\hbox {K}^{-4}$ is the Stefan-Boltzmann constant. The factor $\cos \theta$ is due to the fact that black bodies are Lambertian radiators.

In recent years, several methods have been implemented for achieving a spectrally selective emittance, in particular narrowband emittance, which increases the coherence of the emitted photons. One possibility is to use a material that exhibits optical resonances due to the band structure or due to confinement of the charge carriers^1^. Another method is to use structural optical resonances to enhance and/or suppress the emittance. Recently, photonic crystal structures have been used to implement passive pass band filters that reflect the thermal emission at wavelengths that match the photonic bandgap^2,3^. Alternatively, a truncated photonic crystal can be used to enhance the emittance at resonant frequencies^4,5^.

Recent experiments have shown that it is possible to generate infrared (IR) emission by means of Joule heating created by means of a bias voltage applied to graphene on a $\hbox {SiO}_2$/Si substrate^6,7^. In order to avoid the breakdown of the graphene sheet at around $T=700$ K, the graphene sheet can be encapsulated between hexagonal boron nitride (h-BN) layers, which remove efficiently the heat from graphene. The top layer protects it from oxidation^8,9^. In this way, the graphene sheet can be heated up to $T=1600$ K^9^, or even above $T=2000$ K^8,10^. Kim et al. and Luo et al. demonstrated broadband visible emission peaked around a wavelength of $\lambda =725$ nm^8,9^. By using a photonic crystal substrate made of Si, Shiue et al. demonstrated narrowband near-IR emission peaked at around $\lambda =1600$ nm with an emittance of around $\epsilon =0.07$^10^. To the best of our knowledge, there are neither theoretical nor experimental studies on spectrally selective thermal emission from graphene in the mid-IR range.

Here, we present the proof of concept of a method to tune the spectrally selective thermal emission from nanopatterned graphene (NPG) by means of a gate voltage that varies the resonance wavelength of localized surface plasmons (LSPs) around the circular holes that are arranged in a hexagonal or square lattice pattern in a single graphene sheet in the wavelength regime between 3 and 12 $\upmu$m. By generalizing Planck’s radiation theory to grey-body emission, we show that the thermal emission spectrum can be tuned in or out of the two main atmospheric transparency windows of 3 to 5 $\upmu$m and 8 to 12 $\upmu$m in the mid-IR regime, and also in or out of the opaque mid-IR regime between 5 and 8 $\upmu$m. In addition, the gate voltage can be used to tune the direction of the thermal emission due to the coherence between the localized surface plasmons (LSPs) around the holes due to the nonlocal response function in graphene, which we show by means of a nonlocal fluctuation-dissipation theorem. The main element of the nanostructure is a circular hole of diameter *a* in a graphene sheet. Therefore let us focus first on the optoelectronic properties of a single hole.

**Figure 1:**
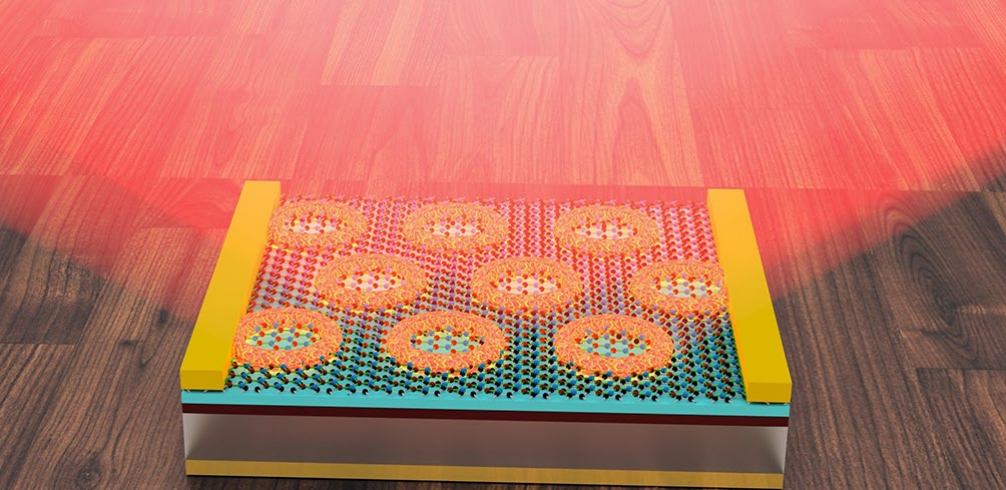
Schematic showing our proposed ultrafast mid-IR light source based on patterned graphene placed on top of a cavity, which can be tuned by means of a gate voltage applied to the ITO layer.

## LSP of a hole in graphene

The frequency-dependent dipole moment of the hole is

4 $$\begin{aligned} \mathbf{p}(\mathbf{r},\omega )& = {} -\varepsilon _0\varepsilon _{||}(\mathbf{r},\omega )\mathbf{E}_{0||} \nonumber \\& = {} -\alpha _{1,2}(\mathbf{r},\omega )\mathbf{E}_{0||}, \end{aligned}$$

where the polarizabilities $\alpha _{1,2}$ are given along the main axes *x* and *y* of the elliptic hole, and $\mathbf{r}=\mathbf{r}_0$ is the position of the dipole moment, i.e. the hole. Graphene’s dielectric function is isotropic in the xy-plane, i.e. $\varepsilon ''_{||}=\varepsilon ''_{xx}=\varepsilon ''_{yy}$. $V_0$ is the volume of the graphene sheet. In the Supplementary Information we derive the general polarizabilities of an uncharged single-sheet hyperboloid with dielectric function $\varepsilon (\omega )$ inside a medium with dielectric constant $\varepsilon _m$ [see Eq. (163)]. The polarizabilities of an elliptical wormhole in *x*- and *y*-direction read

5 $$\begin{aligned} \alpha _1(\omega )& = {} \frac{2abd\pi (\pi /2-1)}{3}\frac{\varepsilon _{||}(\omega )-\varepsilon _m}{\varepsilon _m+L_1[\varepsilon _{||}(\omega )-\varepsilon _m]}, \end{aligned}$$

6 $$\begin{aligned} \alpha _2(\omega )& = {} \frac{2abd\pi (\pi /2-1)}{3}\frac{\varepsilon _{||}(\omega )-\varepsilon _m}{\varepsilon _m+L_2[\varepsilon _{||}(\omega )-\varepsilon _m]}. \end{aligned}$$

respectively, for which the in-plane polarizabilities lie in the plane of the graphene sheet that is parallel to the *xy*-plane. *a* and *b* are the length and the width of the elliptical wormhole, as shown in Fig. 11 in the Supplementary Information. $\varepsilon _{||}(\omega )$ is the dielectric function of graphene. We assumed that the thickness *d* of the graphene sheet is much smaller than the size of the elliptic hole. The geometrical factors in this limit are

7 $$\begin{aligned} L_1\approx & {} abd\int \limits _{\eta _1}^\infty \frac{d\eta '}{(\eta '+a^2)R_{\eta '}}, \end{aligned}$$

8 $$\begin{aligned} L_2\approx & {} abd\int \limits _{\eta _1}^\infty \frac{d\eta '}{(\eta '+b^2)R_{\eta '}}. \end{aligned}$$

In the case of a circular hole of diameter *a* the polarizability simplifies to

9 $$\begin{aligned} \alpha _{||}(\omega ) = \frac{2a^2d\pi (\pi /2-1)}{3}\frac{\varepsilon _{||}(\omega )-\varepsilon _m}{\varepsilon _m+L_{||}[\varepsilon _{||}(\omega )-\varepsilon _m]}, \end{aligned}$$

The localized surface plasmon resonance (LSP) frequency of the hole can be determined from the equation

10 $$\begin{aligned} \varepsilon _m+L_{||}[\varepsilon _{||}(\omega )-\varepsilon _m]=0, \end{aligned}$$

the condition for which the denominator of $\alpha _{||}$ vanishes.

**Figure 2:**
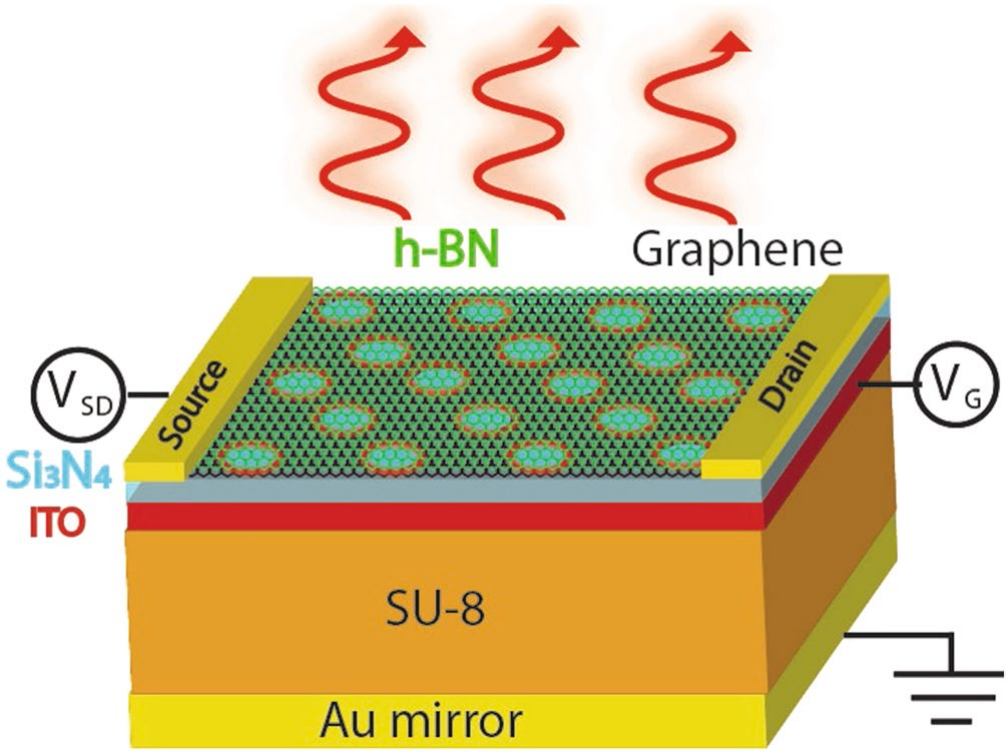
Schematic showing our proposed ultrafast mid-IR light source with the materials used in our setup. The materials from top to bottom are: one single layer of hexagonal boron nitride (h-BN), for preventing oxidation of graphene at higher temperatures, one single layer of patterned graphene, 50 nm of $\hbox {Si}_3\,\hbox {N}_4$, for large n-doping and gating, 50 nm of ITO, metallic contact for gating, which is also transparent in mid-IR, $\lambda /4n_{\mathrm{SU-8}}$ of SU-8^11^, which is transparent in mid-IR, and Au back mirror. $n_{\mathrm{SU-8}}=1.56$ is the refractive index of SU-8.

Using the linear dispersion relation, the intraband optical conductivity is^11,12^

11 $$\begin{aligned} \sigma _{\mathrm{intra}}(\omega ) = \frac{e^2}{\pi \hbar ^2}\frac{2k_BT}{\tau ^{-1} - i\omega }\ln \left[ 2\cosh \left( \frac{\varepsilon _F}{2k_BT} \right) \right] , \end{aligned}$$

which in the case of ${\varepsilon _F} \gg {k_B}T$ is reduced to

12 $$\begin{aligned} \sigma _{\mathrm{intra}}(\omega ) = \frac{e^2}{\pi \hbar ^2}\frac{E_F}{\tau ^{-1} - i\omega }=\frac{2\varepsilon _m\omega _p^2}{\pi \hbar ^2(\tau ^{-1}-i\omega )}, \end{aligned}$$

where $\tau$ is determined by impurity scattering and electron-phonon interaction ${\tau ^{ - 1}} = \tau _{imp}^{ - 1} + \tau _{e - ph}^{ - 1}$ . Using the mobility $\mu$ of the NPG sheet, it can be presented in the form $\tau ^{-1}=ev_F^2/(\mu E_F)$, where $v_F=10^6$ m/s is the Fermi velocity in graphene. $\omega _p=\sqrt{e^2E_F/2\varepsilon _m}$ is the bulk graphene plasma frequency.

**Figure 3:**
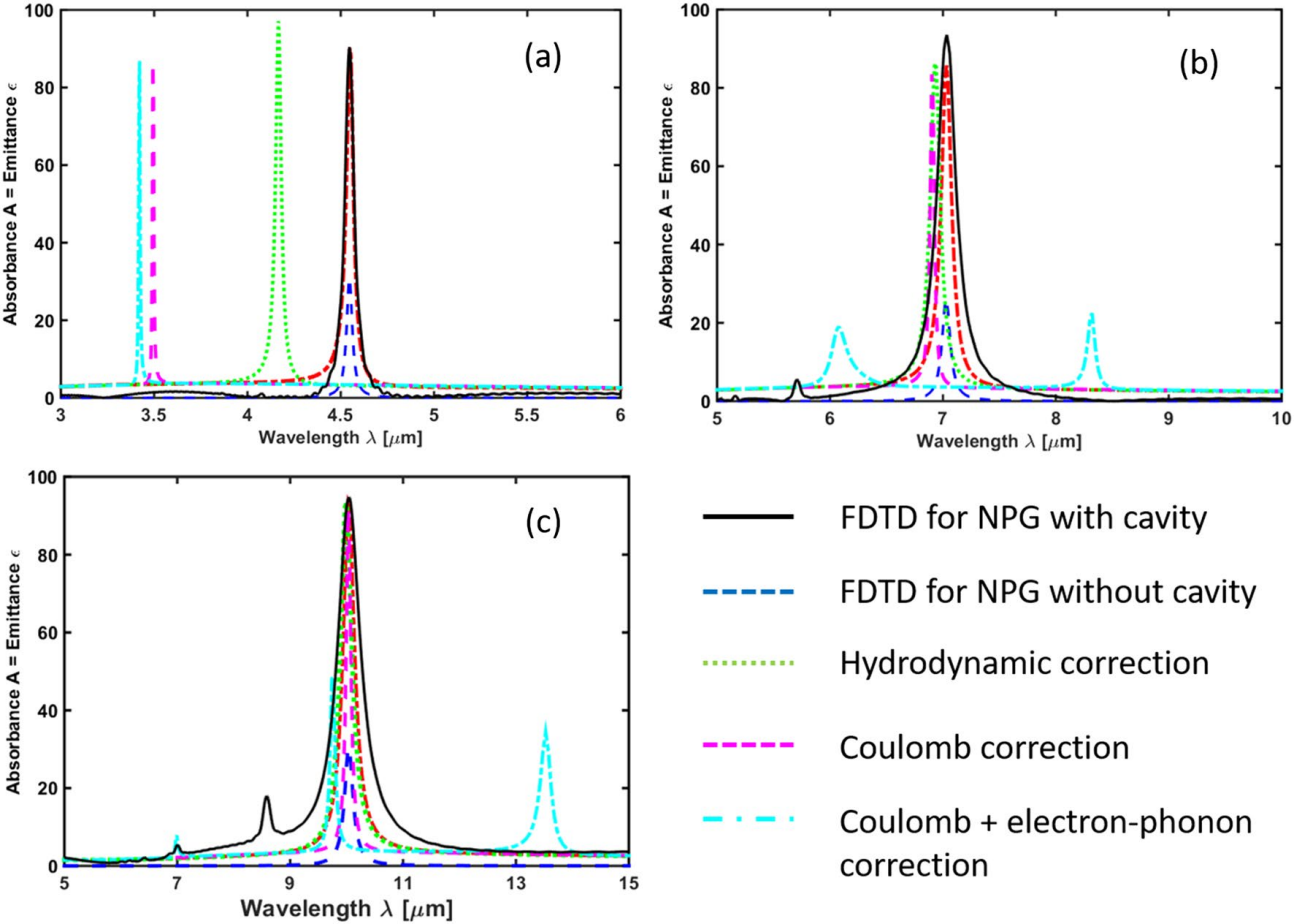
Emittance $\epsilon (\lambda )$ [equal to absorbance $A(\lambda )$] of the structure shown in Figs. 1 and 2 with Fermi energy $E_F=1.0$ eV, mobility $\mu =3000$ V/$\hbox {cm}^2$s, hole diameter of (**a**) $a=30$ nm, (**b**) $a=90$ nm, (**c**) $a=300$ nm, and period (**a**) ${\mathcal {P}}=45$ nm, (**b**) ${\mathcal {P}}=150$ nm, (**c**) ${\mathcal {P}}=450$ nm at $T=300$ K. The solid (black) curve represents the result of FDTD calculation. The dashed (blue) curve and the solid (black) curve are the emittances $\epsilon _g$ and $\epsilon _{\mathrm{FP}}$ calculated by means of Eq. (26) and Eq. (31) for the bare NPG sheet without cavity and the NPG including cavity, respectively. The dotted (green) line exhibits a blue-shift due to the hydrodynamic correction shown in Eq. (13) with $D(\nu =30$ THz$)\approx 0$. The blue-shifted dashed (magenta) curve and the blue-shifted dot-dashed (cyan) curve are the RPA-corrected LSP peaks due to the Coulomb interaction and the Coulomb interaction including electron-phonon interaction with the optical phonons of graphene, boron nitride, and $\hbox {Si}_3\,\hbox {N}_4$. This NPG sheet emits (**a**) into the atmospheric transparency window between 3 and 5 $\upmu$m, (**b**) into the atmosperic opacity window between 5 and 8 $\upmu$m, and (**c**) into the atmospheric transparency window between 8 and 12 $\upmu$m.

It is well-known by now that hydrodynamic effects play an important role in graphene because the Coulomb interaction collision rate is dominant, i.e. $\tau _{ee}^{ - 1}\gg \tau _{imp}^{ - 1}$ and $\tau _{ee}^{ - 1}\gg \tau _{e - ph}^{ - 1}$, which corresponds to the hydrodynamic regime. $\tau _{imp}^{ - 1}$ and $\tau _{e - ph}^{ - 1}$ are the electron-impurity and electron-phonon collision rates, respectively. Since for large absorbance and emittance, we choose a large Fermi energy, we are in the Fermi liquid regime of the graphene sheet. Taking the hydrodynamic correction into account, we also consider the hydrodynamically adjusted intraband optical conductivity^13,14^,

13 $$\begin{aligned} \sigma _{\mathrm{intra}}^{\mathrm{HD}}(\omega )=\frac{\sigma _{\mathrm{intra}}(\omega )}{1-\eta ^2\frac{k_{||}^2}{\omega ^2}}, \end{aligned}$$

where $\eta ^2=\beta ^2+D^2\omega (\gamma +i\omega )$, $\beta ^2\approx \frac{3}{4}v_F^2$ is the intraband pressure velocity, $D\approx 0.4$ $\upmu$m is the diffusion length in graphene, and $\gamma =\tau ^{-1}$ is the relaxation rate. Interestingly, the optical conductivity becomes *k*-dependent and nonlocal. Also, below we will conjecture that the diffusion length *D* must be frequency-dependent. The effect of the hydrodynamic correction on the LSP resonances at around $\lambda =4$ $\upmu$m, 7 $\upmu$m, and 10 $\upmu$m is shown in Fig. 3a–c, respectively.

Note that since $\varepsilon =1+\chi$, where $\chi$ is the susceptibility, it is possible to replace $\varepsilon ''=\chi ''$. Alternatively, using the formula of the polarizability $\alpha =\varepsilon _0\chi$ we can write $\varepsilon ''=\alpha ''/\varepsilon _0$. The dielectric function for graphene is given by^11,12^

14 $$\begin{aligned} \varepsilon _{||}(\omega )=\varepsilon _g+\frac{i\sigma _{2D}(\omega )}{\varepsilon _0\omega d}, \end{aligned}$$

where $\epsilon _g=2.5$ is the dielectric constant of graphite and *d* is the thickness of graphene. Inserting this formula into Eq. (10) gives

15 $$\begin{aligned} \varepsilon _m+L_{||}[\varepsilon _g+ i\frac{e^2}{\pi \hbar ^2}\frac{E_F}{\varepsilon _0\omega d(\tau ^{-1}-i\omega )}-\varepsilon _m]=0, \end{aligned}$$

Solving for the frequency and using the real part we obtain the LSP frequency,

16 $$\begin{aligned} \mathrm{Re}\omega _{\mathrm{LSP}} = \frac{{2L_{||}^2\varepsilon _m\omega _p^2\tau }}{{\pi \hbar ^2 \left\{ {{L^2} + d^2\varepsilon _0^2{{\left[ {L_{||}\left( {\varepsilon _g - \varepsilon _m} \right) +\varepsilon _m} \right] }^2}} \right\} }}, \end{aligned}$$

which is linear in the Fermi energy $E_F$.

## 2D array of holes in graphene

Let us now consider the 2D array of circular holes in a graphene sheet. Since the dipole moments $p_j=\delta \mathbf{p}(\mathbf{R}_j,\omega )$ interact with each other by inducing dipole moments, we need to consider the dressed dipole moment at each site $\mathbf{R}_j$ as source of the electric field, which is

17 $$\begin{aligned} {\tilde{p}}_j =p_j+ \alpha \sum \limits _{j'\ne j}{\mathcal {G}}_{jj'}{\tilde{p}}_{j'}, \end{aligned}$$

where ${\mathcal {G}}_{jj'}$ is the dipole-dipole interaction tensor. Using Bloch’s theorem $p_j=p_0\exp (i\mathbf{k}_{||}\cdot \mathbf{R}_{||})$, the effective dipole moment becomes

18 $$\begin{aligned} {\tilde{p}}_0 =p_0+{\tilde{p}}_0\alpha \sum \limits _{j'\ne j}{\mathcal {G}}_{jj'}e^{i\mathbf{k}_{||}\cdot (\mathbf{R}_j-\mathbf{R}_{j'})}. \end{aligned}$$

for each site *j*, and thus

19 $$\begin{aligned} {\tilde{p}}_0=\frac{p_0}{1-\alpha {\mathcal {G}}}. \end{aligned}$$

The lattice some over the dipole-dipole interaction tensor ${\mathcal {G}}=\sum \limits _{j'\ne j}{\mathcal {G}}_{jj'}e^{i\mathbf{k}_{||}\cdot (\mathbf{R}_j-\mathbf{R}_{j'})}$ can be found in Ref.^15^, i.e.

20 $$\begin{aligned} \mathrm{Re}{\mathcal {G}}\approx & {} g/{\mathcal {P}}^3 , \end{aligned}$$

21 $$\begin{aligned} \mathrm{Im}{\mathcal {G}}& = {} S-2k^3/3, \end{aligned}$$

where ${\mathcal {P}}$ is the lattice period,

22 $$\begin{aligned} S=\frac{2\pi k}{\Omega _0}\times \left\{ \begin{array}{ll} \arccos \theta &{} \text{ for } \text{ s } \text{ polarization, } \\ \cos \theta &{} \text{ for } \text{ p } \text{ polarization. } \end{array} \right. , \end{aligned}$$

$\Omega _0$ is the unit-cell area, and the real part is valid for periods much smaller than the wavelength. The factor $g=5.52$ ($g=4.52$) for hexagonal (square) lattice. The electric field created by the effective dipole moment is determined by

23 $$\begin{aligned} \tilde{\mathbf{p}}_0={\tilde{\alpha }}\mathbf{E}, \end{aligned}$$

from which we obtain the effective polarizability of a hole in the coupled dipole approximation (CDA),

24 $$\begin{aligned} {\tilde{\alpha }}=\frac{\alpha }{1-\alpha {\mathcal {G}}}. \end{aligned}$$

This formula is the same as in Refs.^15,16^, where the absorption of electromagnetic waves by arrays of dipole moments and graphene disks was considered. Thus, our result corroborates Kirchhoff’s law (see below). Consequently, we obtain the same reflection and transmission amplitudes as in Ref.^15^, i.e.

25 $$\begin{aligned} r = \frac{\pm iS}{\alpha ^{-1}-{\mathcal {G}}}, \; t = 1+r, \end{aligned}$$

where the upper (lower) sign and $S=2\pi \omega /c\Omega _0\cos \theta$ ($S=2\pi \omega \cos \theta /c\Omega _0$) apply to s (p) polarization. Thus, the emittance and absorbance of the bare NPG sheet are given by^15,17,18^

26 $$\begin{aligned} \epsilon _g=A_g=1-|r|^2-|t|^2. \end{aligned}$$

The coupling to the interface of the substrate with reflection and transmission amplitudes $r_0$ and $t_0$, respectively, which is located basically at the same position as the NPG sheet, yields the combined reflection and transmission amplitudes^15^

27 $$\begin{aligned} R=r+\frac{tt'r_0}{1-r_0r'}, \; T=\frac{tt_0}{1-r_0r'}, \end{aligned}$$

where $r'=r$ and $t'=1-r$ are the reflection and transmission amplitudes in backwards direction, respectively. The results for the LSP resonances at around $\lambda =4$ $\upmu$m, 7 $\upmu$m, and 10 $\upmu$m are shown in Fig. 3a–c, respectively.

If we include also the whole substrate including cavity and Au mirror, we need to sum over all possible optical paths in the Fabry-Perot cavity, yielding

28 $$\begin{aligned} R_{\mathrm{FP}}=R+TT'r_{\mathrm{Au}}e^{i\delta }\sum \limits _{m=0}^\infty r_m^m, \end{aligned}$$

with

29 $$\begin{aligned} r_m= r_{\mathrm{Au}}R'e^{i\delta }, \end{aligned}$$

where $r_{Au}$ is the complex reflection amplitude of the Au mirror in the IR regime. $\delta =2kL\cos \theta$ is the phase accumulated by one back-and-forth scattering inside the Fabry-Perot cavity of length *L*. $k\approx n_{SU-8}k_0$ is the wavenumber inside the cavity for an external EM wave with wavenumber $k_0=2\pi /\lambda$. Since the sum is taken over a geometric series, we obtain

30 $$\begin{aligned} R_{\mathrm{FP}}=R+\frac{TT'r_{\mathrm{Au}}e^{i\delta }}{1- r_{\mathrm{Au}}R'e^{i\delta }}. \end{aligned}$$

Since the transmission coefficient through the Au mirror can be neglected, we obtain the emittance $\epsilon$ and absorbance *A* including cavity, i.e.

31 $$\begin{aligned} \epsilon _{\mathrm{FP}}=A_{\mathrm{FP}}=1-|R_{\mathrm{FP}}|^2. \end{aligned}$$

The results for the LSP resonances at around $\lambda =4$ $\upmu$m, 7 $\upmu$m, and 10 $\upmu$m are shown in Fig. 3a–c, respectively.

## Spectral radiance of incoherent photons

Using these results, let us consider the excitation of the graphene sheet near the hole by means of thermal fluctuations, which give rise to a fluctuating EM field of a localized surface plasmon (LSP). This can be best understood by means of the fluctuation-dissipation theorem, which provides a relation between the rate of energy dissipation in a non-equilibrium system and the quantum and thermal fluctuations occuring spontaneously at different times in an equilibrium system^19^. The standard (local) fluctuation-dissipation theorem for fluctuating currents $\delta {\hat{J}}_\nu (\mathbf{r},\omega )$ in three dimensions reads

32 $$\begin{aligned} \left<\delta {\hat{J}}_\mu (\mathbf{r},\omega )\delta {\hat{J}}_\nu (\mathbf{r}',\omega ')\right>& = {} \omega \varepsilon _0\varepsilon ''_{\mu \nu }(\mathbf{r},\omega ) \Theta (\omega ) \nonumber \\&\quad \times \delta (\omega -\omega ')\delta (\mathbf{r}-\mathbf{r}'), \end{aligned}$$

where the relative permittivity $\varepsilon (\mathbf{r},\omega )=\varepsilon '(\mathbf{r},\omega )+i\varepsilon ''(\mathbf{r},\omega )=f(\mathbf{r})\varepsilon (\omega )$ and $\mu ,\nu =x,y,z$ are the coordinates. Note that since $\varepsilon =1+\chi$, where $\chi$ is the susceptibility, it is possible to replace $\varepsilon ''=\chi ''$. Alternatively, using the formula of the polarizability $\alpha =\varepsilon _0\chi$ we can write $\varepsilon ''=\alpha ''/\varepsilon _0$. $f(\mathbf{r})=1$ on the graphene sheet and 0 otherwise. Since the fluctuating currents are contained inside the two-dimensional graphene sheet, we write the local fluctuation-dissipation theorem in its two-dimensional form, i.e.

33 $$\begin{aligned} \left<\delta {\hat{J}}_\mu (\mathbf{r}_{||},\omega )\delta {\hat{J}}_\nu (\mathbf{r}_{||}',\omega ')\right>& = {} {\sigma '}_{\mu \nu }^{2D}(\mathbf{r}_{||},\omega ) \Theta (\omega ) \nonumber \\&\quad \times \delta (\omega -\omega ')\delta (\mathbf{r}_{||}-\mathbf{r}_{||}'), \end{aligned}$$

where the fluctuating current densities have units of A/$\hbox {m}^2$ and the coordinates are in-plane of the graphene sheet.

Using the method of dyadic Green’s functions, it is possible to express the fluctuating electric field generated by the fluctuating current density by

34 $$\begin{aligned} \delta {\hat{\mathbf{E}}}(\mathbf{r},\omega )=i\omega \mu _0\int _{\Omega }\mathbf{G}(\mathbf{r},\mathbf{r}_{0||};\omega ) \delta {\hat{\mathbf{J}}}(\mathbf{r}_{0||},\omega )d^2r_{0||}, \end{aligned}$$

where $\Omega$ is the surface of the graphene sheet. The LSP excitation around a hole can be well approximated by a dipole field such that

35 $$\begin{aligned} \delta {\hat{\mathbf{J}}}(\mathbf{r}_{0||},\omega )& = {} -i\omega \sum \limits _j\delta \tilde{\mathbf{p}}(\mathbf{R}_j,\omega ) \nonumber \\& = {} -i\omega \delta \tilde{\mathbf{p}}_0(\omega )\sum \limits _{j}\delta (\mathbf{r}_{0||}-\mathbf{R}_j) , \end{aligned}$$

where $\mathbf{R}_j=(x_j,y_j)$ are the positions of the holes in the graphene sheet.

**Figure 4:**
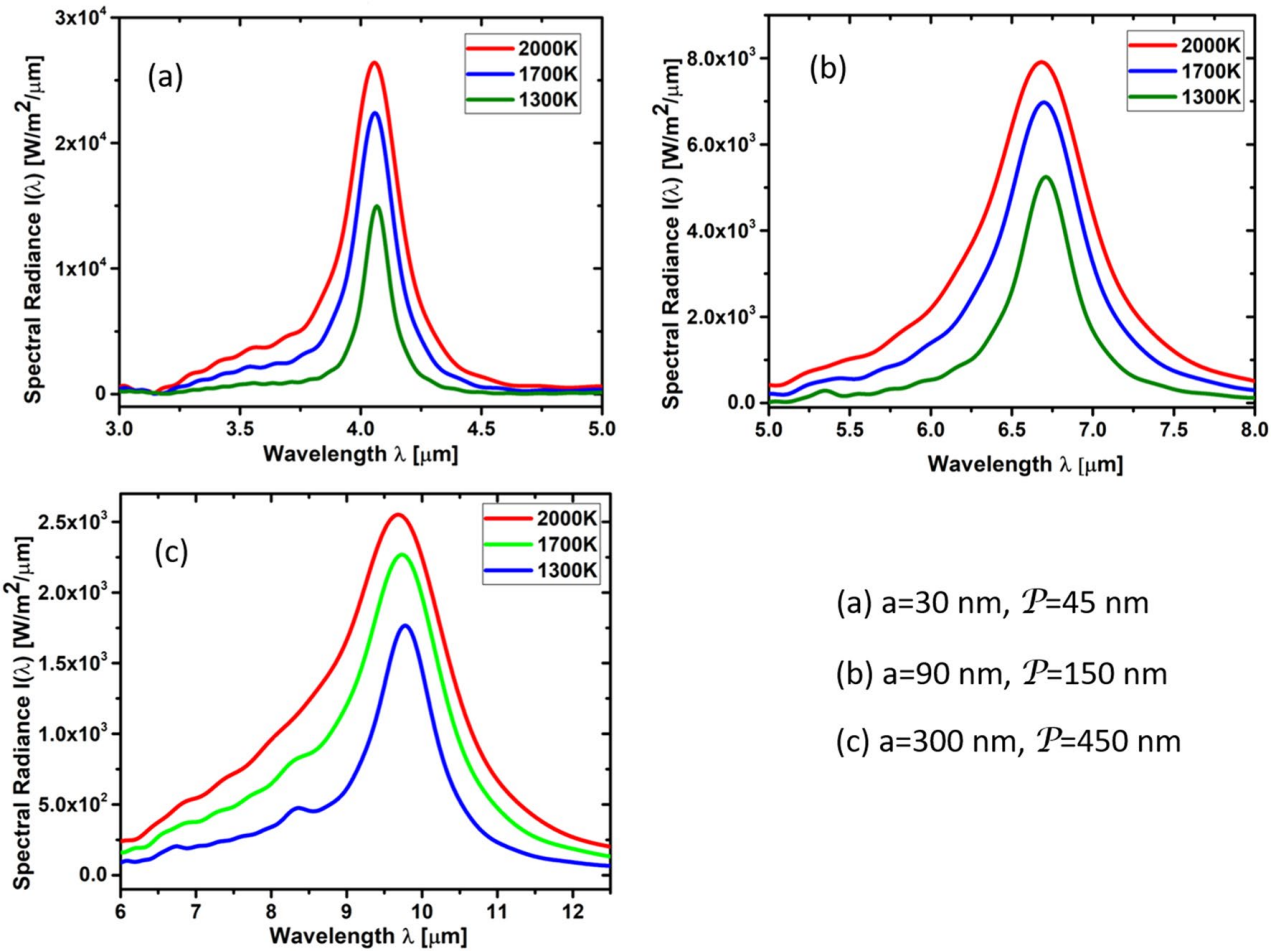
Spectral radiance of NPG including cavity, as shown in in Figs. 1 and 2, as a function of wavelength $\lambda$ with Fermi energy $E_F=1.0$ eV, mobility $\mu =3000$ V/$\hbox {cm}^2$s, hole diameter (**a**) $a=30$ nm, (**b**) $a=90$ nm, (**c**) $a=300$ nm, and period (**a**) ${\mathcal {P}}=45$ nm, (**b**) ${\mathcal {P}}=150$ nm, (**c**) ${\mathcal {P}}=450$ nm at 1300 K, 1700 K, and 2000 K.

Consequently, we have

36 $$\begin{aligned} \delta {\hat{\mathbf{E}}}(\mathbf{r},\omega )=\omega ^2\mu _0\delta \tilde{\mathbf{p}}_0(\omega )\sum \limits _j\mathbf{G}(\mathbf{r},\mathbf{R}_j;\omega ). \end{aligned}$$

The dyadic Green function is defined as

37 $$\begin{aligned} \overleftrightarrow {\mathbf{G}}(\mathbf{r},\mathbf{r}';\omega )=\left[ \overleftrightarrow {\mathbb {1}}+\frac{1}{\mathbf{k}(\omega )^2}{\varvec{\nabla }}{\varvec{\nabla }}\right] G(\mathbf{r},\mathbf{r}';\omega ) \end{aligned}$$

with the scalar Green function given by

38 $$\begin{aligned} G(\mathbf{r},\mathbf{r}';\omega )=\frac{e^{-i\mathbf{k}(\omega )\cdot |\mathbf{r}-\mathbf{r}'|}}{4\pi |\mathbf{r}-\mathbf{r}'|}, \end{aligned}$$

and $$\mathbf{k}(\omega )^2=(\omega ^2/c^2)[\varepsilon _{xx}(\omega ),\varepsilon _{yy}(\omega ),\varepsilon _{zz}(\omega )]$$.

Then, the fluctuation-dissipation theorem can be recast into the form

39 $$\begin{aligned} \left<\delta {\tilde{p}}_\mu (\mathbf{r}_{0||},\omega )\delta {\tilde{p}}_\nu ^*(\mathbf{r}_{0||}',\omega ')\right>& = {} \frac{{\sigma '}_{\mu \nu }^{2D}(\mathbf{R}_i,\omega )}{\omega ^2}\Theta (\omega )\delta (\omega -\omega ') \nonumber \\&\quad \times \delta (\mathbf{r}_{0||}-\mathbf{r}_{0||}'), \end{aligned}$$

and thus we obtain

40 $$\begin{aligned} \left<\delta {\hat{E}}_\mu (\mathbf{r},\omega )\delta {\hat{E}}_\nu ^*(\mathbf{r}',\omega ')\right>& = {} \omega ^4\mu _0^2\sum \limits _{m,m'}\int \limits _{\Omega }d^2r_{0||} G_{\mu m}(\mathbf{r},\mathbf{r}_{0||};\omega ) \nonumber \\&\quad \times \int \limits _{\Omega '}d^2r_{0||}'G_{m'\nu }^*(\mathbf{r}',\mathbf{r}_{0||}';\omega ')\left<\delta {\tilde{p}}_m(\mathbf{r}_0,\omega )\delta {\tilde{p}}_{m'}^*(\mathbf{r}_0',\omega )\right> \nonumber \\& = {} \frac{\omega ^2}{c^4\varepsilon _0^2}\sum \limits _{m}\int \limits _{\Omega }d^2r_{0||}G_{\mu m}(\mathbf{r},\mathbf{r}_{0||};\omega )G_{m'\nu }^*(\mathbf{r}',\mathbf{r}_{0||};\omega ') \nonumber \\&\quad \times \Theta (\omega ){\sigma '}_{mm'}^{2D}(\mathbf{r}_{0||},\omega )\delta (\omega -\omega ') \nonumber \\& = {} \frac{\omega ^2}{c^4\varepsilon _0^2}\sum \limits _{m,j}G_{\mu m}(\mathbf{r},\mathbf{R}_j;\omega )G_{m\nu }^*(\mathbf{r}',\mathbf{R}_j;\omega ') \nonumber \\&\quad \times \Theta (\omega ){\sigma '}_{mm}^{2D}(\mathbf{R}_j,\omega )\delta (\omega -\omega '), \end{aligned}$$

noting that the dielectric tensor $\varepsilon ''(\mathbf{r},\omega )$ is diagonal.

Since the energy density of the emitted electric field at the point $\mathbf{r}$ is

41 $$\begin{aligned} u(\mathbf{r},\omega )\delta (\omega -\omega ')=\varepsilon _0\sum _{i=x,y,z}\left<\delta {\hat{E}}_i^*(\mathbf{r},\omega )\delta {\hat{E}}_i(\mathbf{r},\omega ')\right>, \end{aligned}$$

we can write the spectral radiance as

42 $$\begin{aligned} I(\mathbf{r},\omega )& = {} \frac{\omega ^2}{4\pi c^3\varepsilon _0}\frac{1}{N}\sum \limits _{\mu ; m=x,y;j}\left| G_{\mu m}(\mathbf{r},\mathbf{R}_j;\omega )\right| ^2 \nonumber \\&\quad \times \Theta (\omega ){\sigma '}_{mm}^{2D}(\mathbf{R}_j,\omega ) \nonumber \\& = {} \frac{\omega ^2}{4\pi c^3\varepsilon _0}\Theta (\omega ){\sigma '}_{||}^{2D}(\omega ) \sum \limits _{\mu ,m}\left| G_{\mu m}(\mathbf{r},\mathbf{R}_0;\omega )\right| ^2, \end{aligned}$$

assuming that the dipole current of the LSP is in the plane of the graphene sheet, i.e. the xy-plane, and the polarizability is isotropic, ie. ${\sigma '}_{||}^{2D}={\sigma '}_{xx}^{2D}={\sigma '}_{yy}^{2D}$, and the same for all holes. *N* is the number of holes. In order to obtain the spectral radiance in the far field, we need to integrate over the spherical angle. Using the results from the Supplementary Information, we obtain

43 $$\begin{aligned} I_\infty (\omega )& = {} \frac{\omega ^2\Theta (\omega )}{3\pi ^2\varepsilon _0 c^3}{\sigma '}_{||}^{2D}(\omega ) \nonumber \\& = {} \frac{\omega ^2\Theta (\omega )}{3c^2\pi ^2} A_{||}^{2D}(\omega ), \end{aligned}$$

where we used the definition of the absorbance of a 2D material, i.e.

44 $$\begin{aligned} A_{2D}(\omega )=(1/\varepsilon _0 c)\mathrm{Re}\sigma _{2D}(\omega )=(1/\varepsilon _0 c)\sigma _{2D}'(\omega ), \end{aligned}$$

with 2D complex conductivity $\sigma _{2D}(\omega )$. According to Kirchhoff’s law, emittance $\epsilon (\omega )$, absorbance $A(\omega )$, reflectance $R(\omega )$, and transmittance $T(\omega )$ are related by^20^

45 $$\begin{aligned} \epsilon (\omega )=A(\omega )=1-R(\omega )-T(\omega ), \end{aligned}$$

from which we obtain the grey-body thermal emission formula

46 $$\begin{aligned} I_\infty (\omega ) = \frac{\omega ^2\Theta (\omega )}{3\pi ^2c^2} \epsilon _{||}^{2D}(\omega ), \end{aligned}$$

whose prefactor bears strong similarity to Planck’s black body formula in Eq. (1).

**Figure 5:**
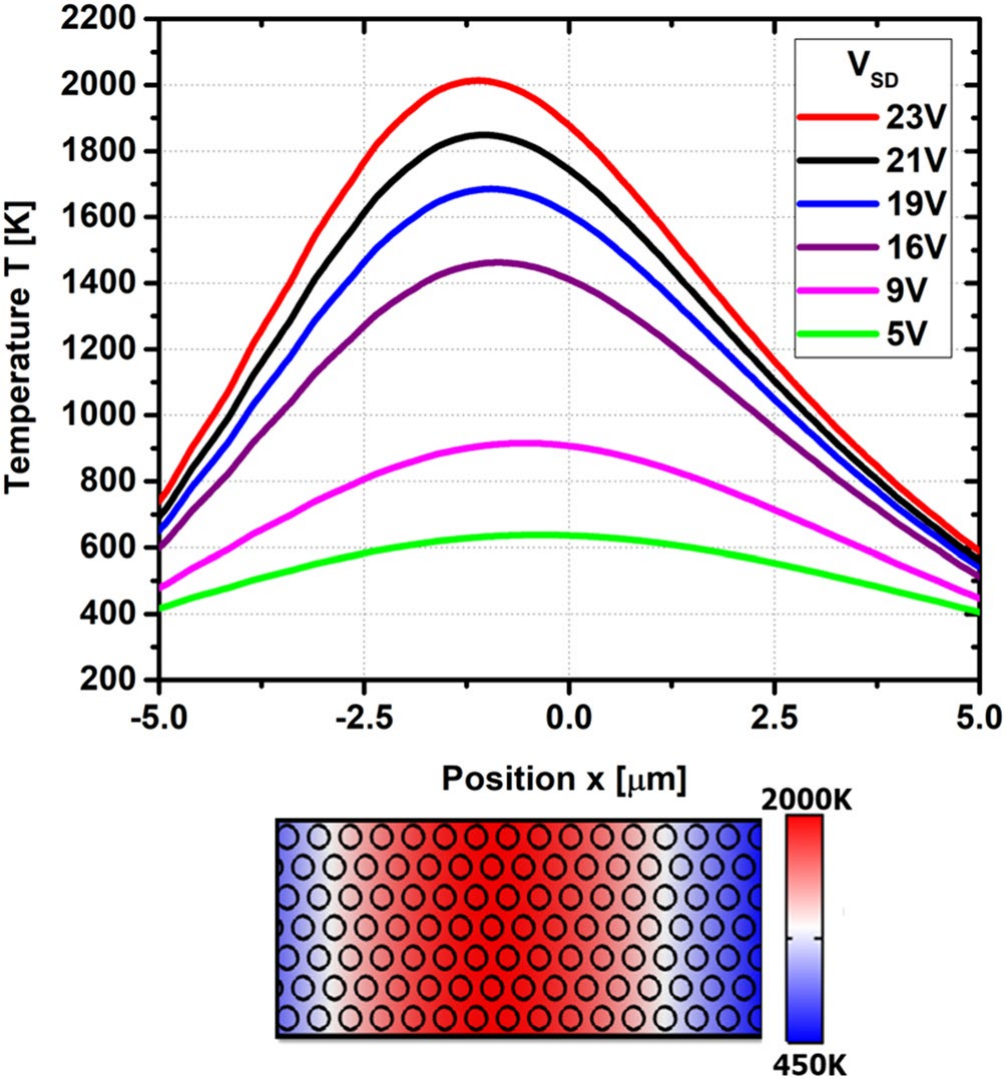
Temperature distribution inside the NPG sheet for various values of the bias voltage $V_{\mathrm{SD}}$, calculated by means of COMSOL. As the bias voltage is increased, the maximum of temperature shifts away from the center of the NPG sheet due to the Peltier effect.

Using FDTD to calculate the emittance $\epsilon _{||}^{2D}(\omega )$, we evaluted the grey-body thermal emission according to Eq. (46) for the thermal emitter structure based on NPG shown in Figs. 1 and 2. Using COMSOL, we calculated the temperature distribution inside the NPG sheet, as shown in Fig. 5, when a bias voltage $V_{\mathrm{SD}}$ is applied, which gives rise to Joule heating. The geometry of the simulated device is shown in Figs. 1 and 2. The area of the graphene channel is 10 $\upmu$m $\times$ 10 $\upmu$m. The thickness of the graphene sheet is 0.5 nm. The size of the gold contacts is 5 $\upmu$m $\times$ 10 $\upmu$m, with a thickness of 50 nm. Our results are shown in Fig. 4a–c for the temperatures 1300 K, 1700 K, and 2000 K of NPG. After integrating over the wavelength under the curves, we obtain the following thermal emission power per area:

**Table:** 

Resonance wavelength	Power per area
4 $\upmu$m	11,221 W/$\hbox {m}^2$
7 $\upmu$m	9820 W/$\hbox {m}^2$
10 $\upmu$m	6356 W/$\hbox {m}^2$

**Figure 6:**
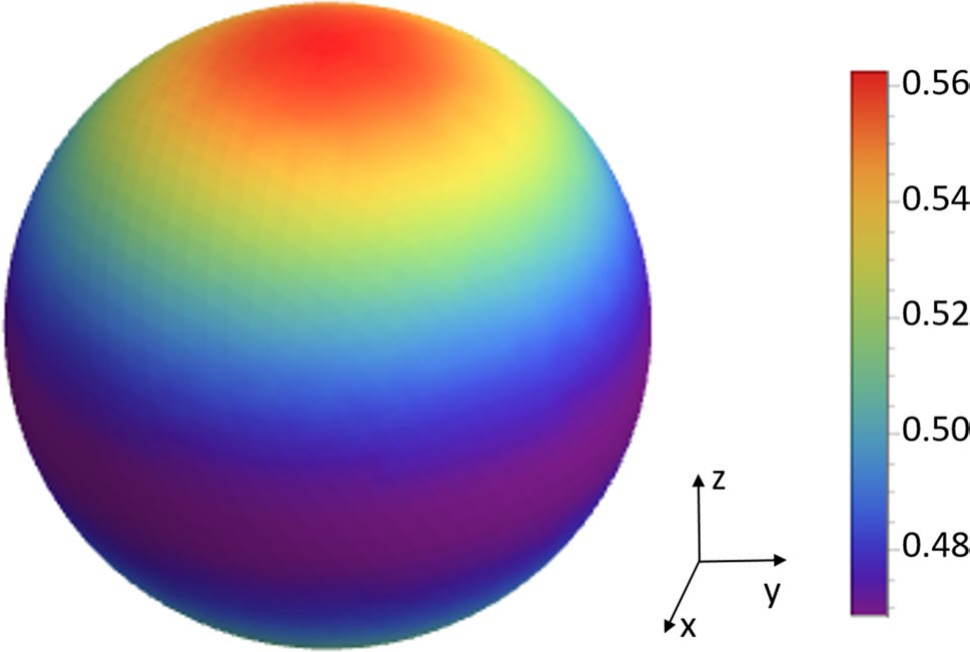
Spherical density plot of the normalized angular intensity distribution ${\hat{I}}(\theta )$ of the thermal emission from NPG in the case of incoherent photons.

Let us consider the dependence of the thermal emission of NPG on the angle $\theta$. Integrating over $r^2\varphi$ we obtain

47 $$\begin{aligned} I(\theta ,\omega )=\frac{\omega ^2}{4\pi c^2}\Theta (\omega )\frac{11+\cos (2\theta )}{16\pi }\epsilon _{||}^{2D}(\omega ), \end{aligned}$$

which is a clear deviation from a Lambert radiator. The pattern of the thermal radiation can be determined by

48 $$\begin{aligned} {\hat{I}}(\theta )& = {} \frac{\int _0^{2\pi }I(\mathbf{r},\omega )r^2 d\varphi }{\int _0^{2\pi }\int _0^{\pi }I(\mathbf{r},\omega )r^2\sin \theta d\theta d\varphi } \nonumber \\& = {} \frac{3}{64}\left[ 11+\cos (2\theta )\right] , \end{aligned}$$

which is shown in Fig. 6. Interestingly, since we assumed that thermal emission is completely incoherent [see Eq. (42)] the thermal emission from NPG is only weakly dependent on the emission angle $\theta$, which can be clearly seen in Fig. 6.

## Partial coherence of plasmons in graphene and the grey-body radiation

However, the assumption that thermal emission of radiation is incoherent is not always true. Since Kirchhoff’s law is valid, thermal sources can be coherent^21^. After theoretical calculations predicted that long-range coherence may exist for thermal emission in the case of resonant surface waves, either plasmonic or phononic in nature^22,23^, experiments showed that a periodic microstructure in the polar material SiC exhibits coherence over many wavelengths and radiates in well-defined and controlled directions^24^. Here we show that the coherence length of a graphene sheet patterned with circular holes can be as large as 150 $\upmu$m due to the plasmonic wave in the graphene sheet, thereby paving the way for the creation of phased arrays made of nanoantennas represented by the holes in NPG.

The coherence of thermal emission can be best understood by means of a nonlocal response function^25^. First, we choose the nonlocal hydrodynamic response function in Eq. (13). Using the 2D version of the fluctuation-dissipation theorem in Eq. (33), we obtain the nonlocal fluctuation-dissipation theorem in the hydrodynamic approximation,

49 $$\begin{aligned} \left<\delta {\hat{J}}_\mu (\mathbf{r}_{||},\omega )\delta {\hat{J}}_\nu (\mathbf{r}_{||}',\omega ')\right>& = {} \sigma _{\mu \nu }^{\mathrm{HD}}(\Delta \mathbf{r}_{||},\omega )\Theta (\omega )\delta (\omega -\omega ') \nonumber \\& = {} \frac{1}{D}\int \limits _0^\infty dk_{||} \frac{\sigma _{\mathrm{intra}}(\omega )e^{-ik_{||}\Delta \mathbf{r}_{||}}}{1-\eta ^2\frac{k_{||}^2}{\omega ^2}} \Theta (\omega )\delta (\omega -\omega ') \nonumber \\& = {} \sigma _{\mathrm{intra}}(\omega ) \frac{\omega \sqrt{\pi /2}}{D\eta }\sin \left( \frac{\omega \Delta \mathbf{r}_{||}}{\eta }\right) \Theta (\omega )\delta (\omega -\omega '), \end{aligned}$$

where $$\Delta \mathbf{r}_{||}=\mathbf{r}_{||}-\mathbf{r}_{||}'$$ and $$\eta ^2=\beta ^2+D^2\omega (\gamma +i\omega )$$. This result suggests that the coherence length is given approximately by *D*, which according to Ref.^13^ would be $$D\approx 0.4$$ $$\upmu$$m. However, the resulting broadening of the LSP resonance peaks would be very large and therefore in complete contradiction to the experimental measurements of the LSP resonance peaks in Refs.^11,26,27^. Thus, we conclude that the hydrodynamic diffusion length must be frequency-dependent with $$D(\nu =0)=0.4$$ $$\upmu$$m. Using the Fermi velocity of $$v_F=10^6$$ m/s and a frequency of $$\nu =30$$ THz, the average oscillation distance is about $$L=v_F\nu ^{-1}=0.033$$ $$\upmu$$m, which is much smaller than $$D(\nu =0)$$ in graphene. Thus we can approximate $$D(\nu =30$$ THz$$)=0$$. We conjecture that there is a crossover for *D* into the hydrdynamic regime when the frequency is reduced below around $$\nu _0=1$$ to 3 THz, below which the hydrodynamic effect leads to a strong broadening of the LSP peaks for NPG. Consequently, the viscosity of graphene should also be frequency-dependent and a crossover for the viscosity should happen at about the same frequency $$\nu _0$$. We plan to elaborate this conjecture in future work. Future experiments could corroborate our conjecture by measuring the absorbance or emittance as a function of wavelength for varying scale of patterning of the graphene sheet.

Next, let us consider the coherence of thermal emission by means of the nonlocal optical conductivity in the RPA approximation. Using the general formula

50 $$\begin{aligned} \sigma (q,\omega )=\frac{ie^2\omega }{q^2}\chi ^0(q,\omega ), \end{aligned}$$

with

51 $$\begin{aligned} \chi ^0(q,\omega )\approx \frac{E_F q^2}{\pi \hbar ^2\omega (\omega +i\tau ^{-1})} \end{aligned}$$

in the low-temperature and low-frequency approximation, one obtains Eq. (12). Now, let us use the full polarization in RPA approximation including only the Coulomb interaction,

52 $$\begin{aligned} \chi ^{\mathrm{RPA}}(q,\omega )=\frac{\chi ^0(q,\omega )}{1- v_c(q){\chi ^0}(q,\omega ) }, \end{aligned}$$

from which we obtain

53 $$\begin{aligned} \sigma ^{\mathrm{RPA}}(q,\omega )& = {} \frac{ie^2\omega }{q^2}\chi (q,\omega ) \nonumber \\& = {} \frac{ie^2\omega E_F}{\pi \hbar ^2\omega (\omega +i\tau ^{-1})-\frac{e^2E_F}{2\epsilon _0} q}, \end{aligned}$$

which introduces the nonlocal response via the Coulomb interaction in the denominator. The effect of the RPA correction on the LSP resonances at around $\lambda =4$ $\upmu$m, 7 $\upmu$m, and 10 $\upmu$m is shown in Fig. 3a–c, respectively. After taking the Fourier transform, we obtain the nonlocal fluctuation-dissipation theorem in RPA approximation,

54 $$\begin{aligned} & \left \langle \delta {\hat{J}}_\mu (\mathbf{r}_{||},\omega )\delta {\hat{J}}_\nu (\mathbf{r}_{||}',\omega ')\right\rangle = \sigma _{\mu \nu }^{\mathrm{RPA}}(\Delta \mathbf{r}_{||},\omega )\Theta (\omega )\delta (\omega -\omega ') \nonumber \\&\quad =\frac{\sqrt{2\pi }\epsilon _0\omega }{C_{RPA}} e^{iK_{RPA}\Delta \mathbf{r}_{||}-\frac{\Delta {\mathbf{r}}_{||}}{C_{RPA}}} \Theta (\omega )\delta (\omega -\omega '), \end{aligned}$$

where the coherence length in RPA approximation is

55 $$\begin{aligned} C_{RPA}=\frac{e^2|E_F|}{2\pi \hbar ^2\epsilon _0\gamma \omega }, \end{aligned}$$

and the coherence wavenumber is given by

56 $$\begin{aligned} K_{RPA}=\frac{2\pi \hbar ^2\epsilon _0\omega ^2}{e^2|E_F|}. \end{aligned}$$

For simplicity, we switch now to a square lattice of holes. In the case of the LSP resonance for a square lattice of holes at $\lambda =10$ $\upmu$m, corresponding to $\nu =30$ THz, $E_F=1.0$ eV, $\omega =2\pi \nu$, and $\gamma =ev_F^2/(\mu E_F)=0.3$ THz for $\mu =3000$ $\hbox {cm}^2\,\hbox {V}^{-1}\,\hbox {s}^{-1}$, which results in a coherence length of $C_{RPA}=3$ $\upmu$m. This result is in reasonable agreement with the full width at half maximum (FWHM) values of the widths of the LSP resonance peaks in Refs.^11,26,27^. This coherence length would allow to preserve coherence for a linear array of period ${\mathcal {P}}=300$ nm and $C_{RPA}/{\mathcal {P}}=10$ holes. In order to show the coherence length that can be achieved with graphene, we can consider a suspended graphene sheet with a mobility of $\mu =150{,}00$ $\hbox {cm}^2\,\hbox {V}^{-1}\,\hbox {s}^{-1}$. Then the coherence length increases to a value of $C_{RPA}=13$ $\upmu$m, which would allow for coherence over a linear array with $C_{RPA}/{\mathcal {P}}=43$ holes.

**Figure 7:**
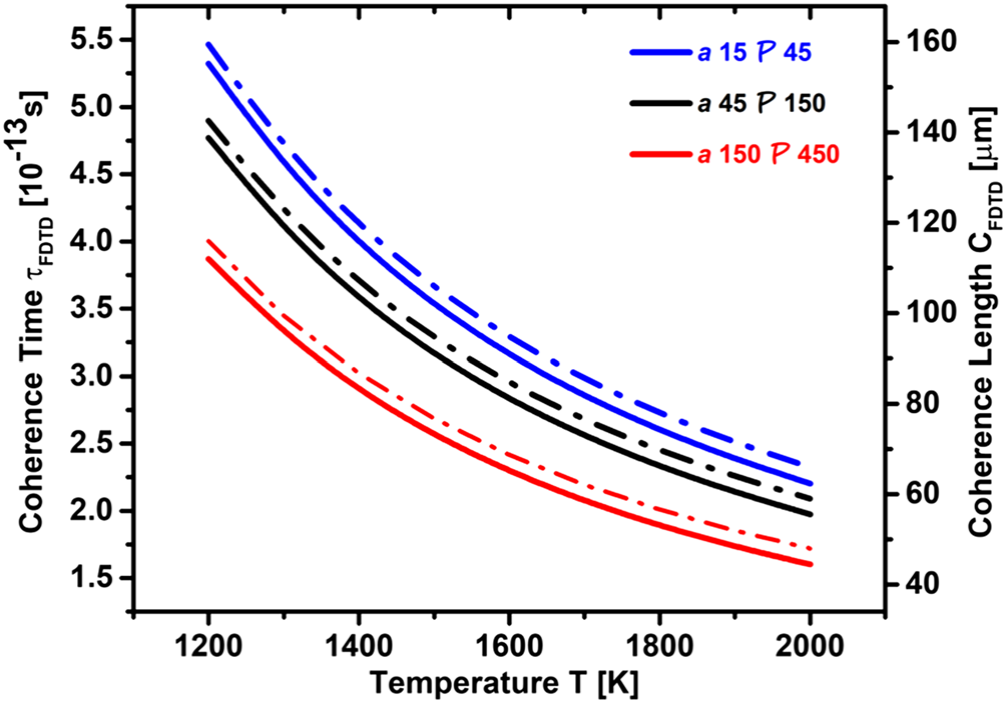
Coherence length $C_{\mathrm{FDTD}}$ and coherence time $\tau _{\mathrm{FDTD}}$ of emitted photons, extracted from the full-width half-maximum (FWHM) of the spectral radiances shown in Fig. 4a–c.

In the case of the LSP resonance for a square lattice of holes at $\lambda =5$ $\upmu$m, corresponding to $\nu =60$ THz, $E_F=1.0$ eV, $\omega =2\pi \nu$, and $\gamma =ev_F^2/(\mu E_F)=0.3$ THz for $\mu =3000$ $\hbox {cm}^2\,\hbox {V}^{-1}\,\hbox {s}^{-1}$ , which results in a coherence length of $C_{RPA}=1.5$ $\upmu$m. Considering again a suspended graphene sheet, the coherence length can be increased to $C_{RPA}=6.7$ $\upmu$m. Since the period in this case is ${\mathcal {P}}=45$ nm, the coherence for $\mu =3000$ $\hbox {cm}^2\,\hbox {V}^{-1}\,\hbox {s}^{-1}$ and $\mu =150{,}00$ $\hbox {cm}^2\,\hbox {V}^{-1}\,\hbox {s}^{-1}$ can be preserved for a linear array of $C_{RPA}/{\mathcal {P}}=33$ and 148 holes, respectively.

The coherence length and time of thermally emitted photons is larger because the photons travel mostly in vacuum. Taking advantage of the Wiener-Kinchine theorem^21^, we can extract the coherence length $C_{\mathrm{FDTD}}$ and coherence time $\tau _{\mathrm{FDTD}}$ of thermally emitted photons by means of the full-width half-maximum (FWHM) of the spectral radiances shown in Fig. 4a–c. Our results are shown in Fig. 7. The coherence length of the thermally emitted photons can reach up to $C_{\mathrm{FDTD}}=150$ $\upmu$m at a resonance wavelength of $\lambda =4$ $\upmu$m. This means that the coherence length of the thermally emitted photons is about 37 times larger than the wavelength.

## Phased array of LSPs in graphene

**Figure 8:**
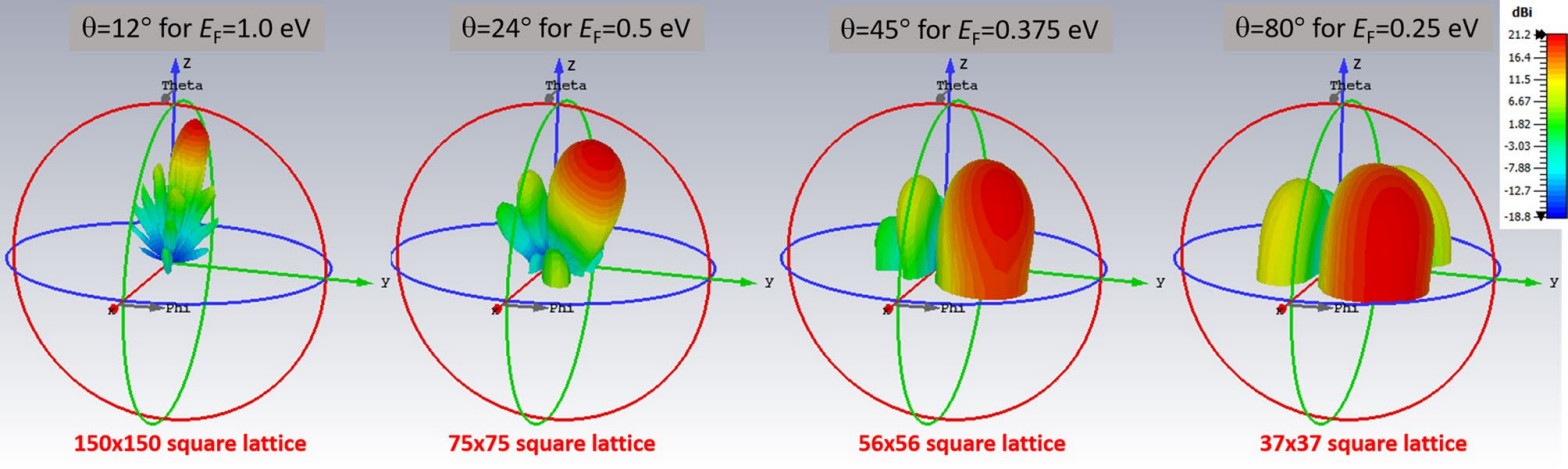
Directivity of the thermal emission from NPG where the holes act as nanoantennas in a phased array. This emission pattern for $E_F=1.0$ eV can be used for surface-emitting mid-IR sources. In the case of a 150x150, 75x75, 56x56, 37x37 square lattice of holes (size of lattice matches coherence length) with period ${\mathcal {P}}=45$ nm and hole diameter of 30 nm, introducing a relative phase of $2.43^\circ$, $4.86^\circ$, $7.28^\circ$, $9.71^\circ$ between the nanoantennas allows for beamsteering in the range between $\theta =12^\circ$ and $\theta =80^\circ$ by tuning the Fermi energy in the range between $E_F=1.0$ eV and $E_F=0.25$ eV.

Thus, the latter large coherence length allows for the coherent control of a 150x150 square array of holes with period ${\mathcal {P}}=45$ nm, individually acting as nanoantennas, that can be used to create a phased array of nanoantennas. One of the intriguing properties of a phased array is that it allows to control the directivity of the emission of photons, which is currently being implemented for large 5G antennas in the 3 to 30 GHz range. The beamsteering capability of our NPG sheet is shown in Fig. 8. In contrast, our proposed phased array based on NPG can operate in the 10 to 100 THz range.

The temporal control of the individual phases of the holes requires an extraordinary fast switching time of around 1 ps, which is not feasible with current electronics. However, the nonlocal response function reveals a spatial phase shift determined by the coherence wavenumber $K_{RPA}$, which is independent of the mobility of graphene. In the case of the LSP resonance at $\lambda =4$ $\upmu$m, we obtain $\lambda _{RPA}=2\pi /K_{RPA}=6$ $\upmu$m, resulting in a minimum phase shift of $2\pi {\mathcal {P}}/\lambda _{RPA}=0.042=2.4^{\circ }$ between neighboring holes, which can be increased to a phase shift of $9.7^{\circ }$ by decreasing the Fermi energy to $E_F=0.25$ eV. Thus, the phase shift between neighboring holes can be tuned arbitrarily between $2.4^{\circ }$ and $9.7^{\circ }$ by varying the Fermi energy between $E_F=1.0$ eV and $E_F=0.25$ eV. Fig. 8 shows the capability of beamsteering for our proposed structure by means of directional thermal emission, which is tunable by means of the gate voltage applied to the NPG sheet.

Due to the full control of directivity with angle of emission between $\theta =12^\circ$ and $\theta =80^\circ$ by tuning the Fermi energy in the range between $E_F=1.0$ eV and $E_F=0.25$ eV, thereby achieving beamsteering by means of the gate voltage, our proposed mid-IR light source based on NPG can be used not only in a vertical setup for surface emission, but also in a horizontal setup for edge emission, which is essential for nanophotonics applications.

## Conclusion

In conclusion, we have demonstrated in our theoretical study that NPG can be used to develop a plasmonically enhanced mid-IR light source with spectrally tunable selective thermal emission. Most importantly, the LSPs along with an optical cavity increase substantially the emittance of graphene from about 2% for pristine graphene to 80% for NPG, thereby outperforming state-of-the-art graphene light sources working in the visible and NIR by at least a factor of 100. Combining our proposed mid-IR light source based on patterned graphene with our demonstrated mid-IR detector based on NPG^27^, we are going to develop a mid-IR spectroscopy and detection platform based on patterned graphene that will be able to detect a variety of molecules that have mid-IR vibrational resonances, such as CO, $\hbox {CO}_2$, NO, $\hbox {NO}_2$, $\hbox {CH}_4$, TNT, $\hbox {H}_2\,\hbox {O}_2$, acetone, TATP, Sarin, VX, etc. In particular, a recent study showed that it is possible to detect the hepatitis B and C viruses label-free at a wavelength of around 6 $\upmu$m^28^. Therefore, we will make great effort to demonstrate that our platform will be able to detect with high sensitivity and selectivity the COVID-19 virus and other viruses that pose a threat to humanity.

## Figures and Tables

**Figure 9:**
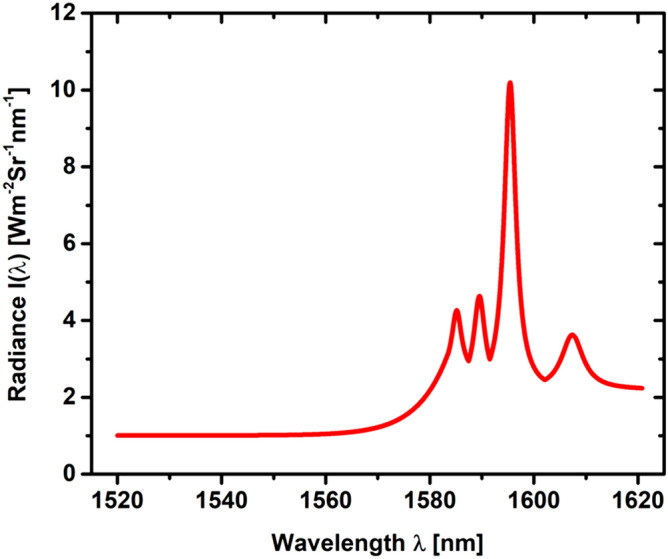
Theoretical fit to spectral radiance presented in Ref.^10^. Shiue et al. used a photonic crystal structure to filter the thermal emission from pristine graphene with an emittance of around $A=0.07$. Integrating the spectral radiance under the curve gives a value of about $P/A=100$ W/$\hbox {m}^2$, which is about 100 times weaker than our proposed thermal radiation source based on NPG.

**Figure 10:**
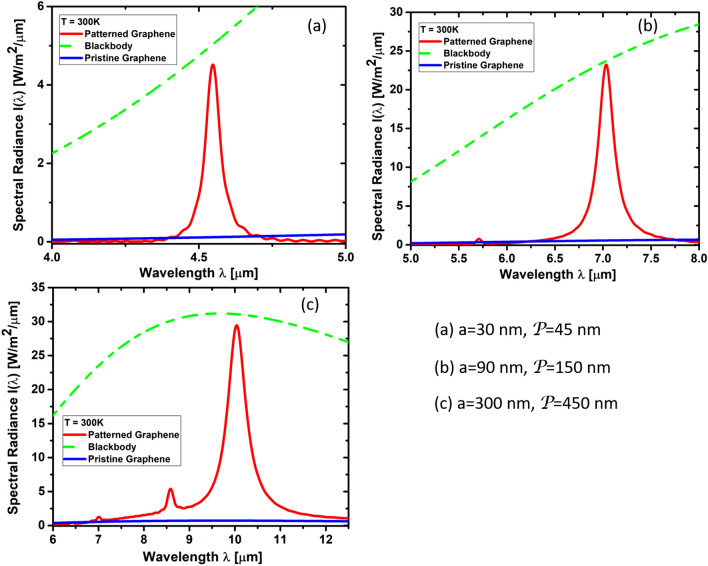
The NPG sheet allows for spectrally selective thermal emission at around (**a**) $\lambda =4.5$ $\upmu$m, (**b**) $\lambda =7$ $\upmu$m, (**c**) $\lambda =10$ $\upmu$m for a period of (**a**) ${\mathcal {P}}=45$ nm, (**b**) ${\mathcal {P}}=150$ nm, (**c**) ${\mathcal {P}}=450$ nm and a hole diameter of (**a**) $a=30$ nm, (**b**) $a=90$ nm, (**c**) $a=300$ nm.

**Figure 11:**
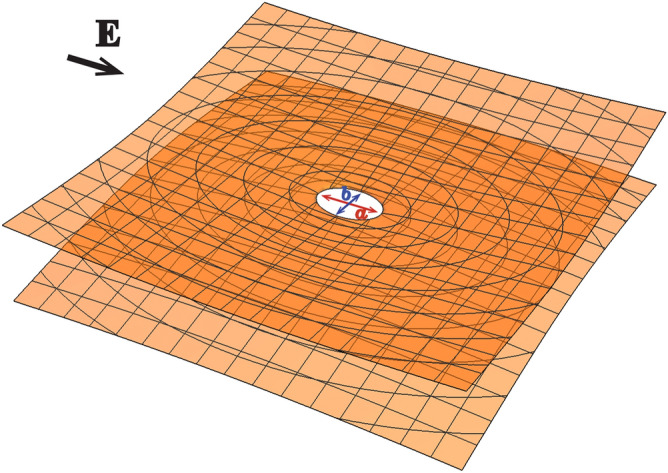
Schematic showing single-sheet hyperboloid with an elliptical wormhole of length *a*, width *b*, and depth $c=0$. The electric field $\mathbf{E}_0$ points along the *a* axis of the ellipse.

**Figure 12:**
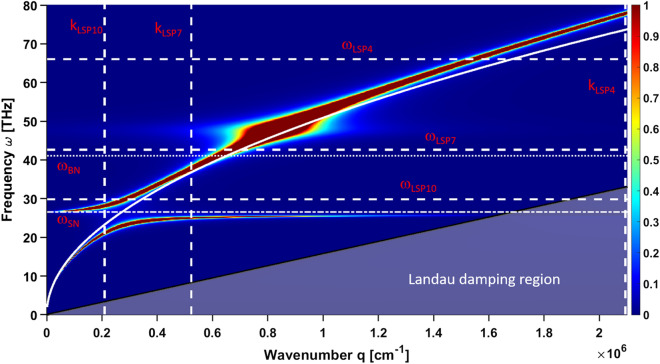
The energy loss function for graphene with $E_F=1.0$ eV. $k_{LSP4}$, $k_{LSP7}$, and $k_{LSP10}$ are the plasmon wavenumbers associated with the nanopatterning of the graphene sheet shown in Fig. 4**a**–**c**, respectively. $\omega _{LSP4}$, $\omega _{LSP7}$, and $\omega _{LSP10}$ represent the LSP resonances shown in Fig. 4a–c, respectively. The polar phonon resonance of h-BN and the surface polar phonon resonance of $\hbox {Si}_3\,\hbox {N}_4$ are denoted by $\omega _{BN}$, and $\omega _{SN}$, respectively. The Landau damping region is marked by the shaded area.

## Appendix

### Spectrally selective thermal emission

Kirchhoff’s law of thermal radiation states that emittance $\epsilon$ is equal to absorbance *A*, i.e.

57 $$\begin{aligned} \epsilon (\omega ,\theta ,\phi ,T)=A(\omega ,\theta ,\phi ,T). \end{aligned}$$

In the case of a black body $\epsilon (\omega ,\theta ,\phi ,T)=A(\omega ,\theta ,\phi ,T)=1$. Pristine graphene has a very small absorbance of only $A=0.023$ and is a nearly transparent body. Shiue et al. used a photonic crystal structure to filter the thermal emission from pristine graphene with an emittance of around $A=0.07$^10^. Their spectral radiance is shown in Fig. 9 and exhibits peaks at around $\lambda =1.55$ $\upmu$m at a temperature of $T=2000$ K. After integrating the spectral radiance under the curve, one obtains a emission power per area of about $P/A=100$ W/$\hbox {m}^2$, which is about 100 times weaker than our proposed thermal radiation source based on NPG at $T=2000$ K. Our proposed thermal mid-IR source features an emission power per area of about $P/A=10^4$ W/$\hbox {m}^2$ at $T=2000$ K. In addition, our proposed thermal mid-IR source features frequency-tunability and beamsteering by means of a gate voltage applied to the NPG sheet.

Using FDTD to calculate the emittance $\epsilon _{||}^{2D}(\omega )$, we evaluted the grey-body thermal emission according to Eq. (46) for the thermal emitter structure based on NPG shown in Figs. 1 and 2. Our results for the temperature $T=300$ K of NPG are shown in Fig. 10a–c. In these figures we compare our results for NPG with the results for pristine graphene and black body radiation.

### Ellipsoidal coordinates

For determining the EM properties of an infinitesimally thin conducting elliptical disk of radius *R* or an infinitesimally thin conducting plane with a elliptical hole, including coated structures, it is most convenient to perform the analytical calculations in the ellipsoidal coordinate system ($\xi$, $\eta$, $\zeta$)^29–32^, which is related to the Cartesian coordinate system through the implicit equation

58 $$\begin{aligned} \frac{x^2}{a^2+u}+\frac{y^2}{b^2+u}+\frac{z^2}{c^2+u}=1 \end{aligned}$$

for $a>b>c$. The cubic roots $\xi$, $\eta$, and $\zeta$ are all real in the ranges

59 $$\begin{aligned} -a^2\le \zeta \le -b^2,\; -b^2\le \eta \le -c^2,\; -c^2\le \xi <\infty , \end{aligned}$$

which are the ellipsoidal coordinates of a point (*x*, *y*, *z*). The surfaces of contant $\xi$, $\eta$, and $\zeta$ are ellipsoids, hyperboloids of one sheet, and hyperboloids of two sheets, respectively, all confocal with the ellipsoid defined by

60 $$\begin{aligned} \frac{x^2}{a^2}+\frac{y^2}{b^2}+\frac{z^2}{c^2}=1. \end{aligned}$$

Each point (*x*, *y*, *z*) in space is determined by the intersection of three surfaces, one from each of the three families, and the three surfaces are orthogonal to each other. The transformation between the two coordinate systems is given by the solutions of Eq. (58), i.e.

61 $$\begin{aligned} x& = {} \pm \sqrt{\frac{(\xi +a^2)(\eta +a^2)(\zeta +a^2)}{(b^2-a^2)(c^2-a^2)}}, \end{aligned}$$

62 $$\begin{aligned} y& = {} \pm \sqrt{\frac{(\xi +b^2)(\eta +b^2)(\zeta +b^2)}{(c^2-b^2)(a^2-b^2)}}, \end{aligned}$$

63 $$\begin{aligned} z& = {} \pm \sqrt{\frac{(\xi +c^2)(\eta +c^2)(\zeta +c^2)}{(a^2-c^2)(b^2-c^2)}}, \end{aligned}$$

defining 8 equivalent octants. The length elements in ellipsoidal coordinates read

64 $$\begin{aligned} dl^2& = {} h_1^2d\xi ^2+h_2^2 d\eta ^2+h_3^2 d\zeta ^2, \end{aligned}$$

65 $$\begin{aligned} h_1& = {} \sqrt{\frac{(\xi -\eta )(\xi -\zeta )}{2R_\xi }}, \end{aligned}$$

66 $$\begin{aligned} h_2& = {} \sqrt{\frac{(\eta -\zeta )(\xi -\zeta )}{2R_\eta }}, \end{aligned}$$

67 $$\begin{aligned} h_3& = {} \sqrt{\frac{(\zeta -\xi )(\zeta -\eta )}{2R_\zeta }}, \end{aligned}$$

68 $$\begin{aligned} R_u^2& = {} (u+a^2)(u+b^2)(u+c^2), \; u=\xi ,\eta ,\zeta . \end{aligned}$$

For the transformation from cartesian to ellipsoidal coordinates, one can use the following system of equations:

69 $$\begin{aligned} \hat{{\varvec{\xi }}}& = {} \frac{\frac{\partial x}{\partial \xi }\hat{\mathbf{x}}+ \frac{\partial y}{\partial \xi }\hat{\mathbf{y}}+ \frac{\partial z}{\partial \xi }\hat{\mathbf{z}}}{\sqrt{\left( \frac{\partial x}{\partial \xi }\right) ^2+\left( \frac{\partial y}{\partial \xi }\right) ^2+\left( \frac{\partial z}{\partial \xi }\right) ^2}} , \end{aligned}$$

70 $$\begin{aligned} \hat{{\varvec{\eta }}}& = {} \frac{\frac{\partial x}{\partial \eta }\hat{\mathbf{x}}+ \frac{\partial y}{\partial \eta }\hat{\mathbf{y}}+ \frac{\partial z}{\partial \eta }\hat{\mathbf{z}}}{\sqrt{\left( \frac{\partial x}{\partial \eta }\right) ^2+\left( \frac{\partial y}{\partial \eta }\right) ^2+\left( \frac{\partial z}{\partial \eta }\right) ^2}} , \end{aligned}$$

71 $$\begin{aligned} \hat{{\varvec{\zeta }}}& = {} \frac{\frac{\partial x}{\partial \zeta }\hat{\mathbf{x}}+ \frac{\partial y}{\partial \zeta }\hat{\mathbf{y}}+ \frac{\partial z}{\partial \zeta }\hat{\mathbf{z}}}{\sqrt{\left( \frac{\partial x}{\partial \zeta }\right) ^2+\left( \frac{\partial y}{\partial \zeta }\right) ^2+\left( \frac{\partial z}{\partial \zeta }\right) ^2}}, \end{aligned}$$

whose elements $J_{ij}$ define the Jacobian matrix. The derivatives are explicitly:

72 $$\begin{aligned} \frac{\partial x}{\partial \xi }& = {} \frac{1}{2}\sqrt{\frac{(a^2+\eta )(a^2+\zeta )}{(a^2+\xi )(a^2-b^2)(a^2-c^2)}} , \end{aligned}$$

73 $$\begin{aligned} \frac{\partial x}{\partial \eta }& = {} \frac{1}{2}\sqrt{\frac{(a^2+\xi )(a^2+\zeta )}{(a^2+\eta )(a^2-b^2)(a^2-c^2)}} , \end{aligned}$$

74 $$\begin{aligned} \frac{\partial x}{\partial \zeta }& = {} \frac{1}{2}\sqrt{\frac{(a^2+\xi )(a^2+\eta )}{(a^2+\zeta )(a^2-b^2)(a^2-c^2)}} , \end{aligned}$$

75 $$\begin{aligned} \frac{\partial y}{\partial \xi }& = {} \frac{1}{2}\sqrt{\frac{(b^2+\eta )(b^2+\zeta )}{(b^2+\xi )(b^2-a^2)(b^2-c^2)}} , \end{aligned}$$

76 $$\begin{aligned} \frac{\partial y}{\partial \eta }& = {} \frac{1}{2}\sqrt{\frac{(b^2+\xi )(b^2+\zeta )}{(b^2+\eta )(b^2-a^2)(b^2-c^2)}} , \end{aligned}$$

77 $$\begin{aligned} \frac{\partial y}{\partial \zeta }& = {} \frac{1}{2}\sqrt{\frac{(b^2+\xi )(b^2+\eta )}{(b^2+\zeta )(b^2-a^2)(b^2-c^2)}} , \end{aligned}$$

78 $$\begin{aligned} \frac{\partial z}{\partial \xi }& = {} \frac{1}{2}\sqrt{\frac{(c^2+\eta )(c^2+\zeta )}{(c^2+\xi )(c^2-a^2)(c^2-b^2)}} , \end{aligned}$$

79 $$\begin{aligned} \frac{\partial z}{\partial \eta }& = {} \frac{1}{2}\sqrt{\frac{(c^2+\xi )(c^2+\zeta )}{(c^2+\eta )(c^2-a^2)(c^2-b^2)}} , \end{aligned}$$

80 $$\begin{aligned} \frac{\partial z}{\partial \zeta }& = {} \frac{1}{2}\sqrt{\frac{(c^2+\xi )(c^2+\eta )}{(c^2+\zeta )(c^2-a^2)(c^2-b^2)}}. \end{aligned}$$

The coordinate $\eta$ is constant on the surfaces of oblate spheroids defined by

81 $$\begin{aligned} \frac{x^2+y^2}{(R\cosh \eta )^2}+\frac{z^2}{(R\sinh \eta )^2}=1 \end{aligned}$$

The surface associated with the limit $\eta \rightarrow 0$ is an infinitesimally thin circular disk of radius *R*. In contrast, the surface in the limit $\eta \gg 1$ is a sphere of radius $r=R\cosh \eta \approx R\sinh \eta$. Thus, the Laplace equation in ellipsoidal coordinates reads

82 $$\begin{aligned} \Delta \Phi&= \frac{4}{(\xi -\eta )(\zeta -\xi )(\eta -\zeta )} \left[ (\eta -\zeta )R_\xi \frac{\partial }{\partial \xi }\left( R_\xi \frac{\partial \Phi }{\partial \xi }\right) \right. \nonumber \\&\quad +\left. (\zeta -\xi )R_\eta \frac{\partial }{\partial \eta }\left( R_\eta \frac{\partial \Phi }{\partial \eta }\right) +(\xi -\eta )R_\zeta \frac{\partial }{\partial \zeta }\left( R_\zeta \frac{\partial \Phi }{\partial \zeta }\right) \right] =0. \end{aligned}$$

### Charged conducting ellipsoid

The surface of the conducting ellipsoid is defined by $\xi =0$. Thus, the electric field potential $\Phi (\xi )$ is a function of $\xi$ only, thereby defining the equipotential surfaces by confocal ellipsoids. Laplace’s equation is then simplified to

83 $$\begin{aligned} \frac{d}{d\xi }\left( R_\xi \frac{d\Phi }{d\xi }\right) =0. \end{aligned}$$

The solution outside the ellipsoid is

84 $$\begin{aligned} \Phi _{\mathrm{out}}(\xi )=A\int \limits _\xi ^\infty \frac{d\xi '}{R_{\xi '}}. \end{aligned}$$

From the asymptotic approximation $\xi \approx r^2$ for large distances $r\rightarrow \infty$, i.e. $\xi \rightarrow \infty$, we identify $R_\xi \approx \xi ^{3/2}$ and thus

85 $$\begin{aligned} \Phi _{\mathrm{out}}(\xi \rightarrow \infty )\approx \frac{2A}{\sqrt{\xi }}=\frac{2A}{r}. \end{aligned}$$

using the boundary condition $\lim _{\xi \rightarrow \infty }\Phi (\xi )=0$. Since the Coulomb field should be $\Phi (\xi \rightarrow \infty )\approx e/r$ at large distances from the ellipsoid, $2A=e$ and

86 $$\begin{aligned} \Phi _{\mathrm{out}}(\xi )=\frac{e}{2}\int \limits _\xi ^\infty \frac{d\xi '}{R_{\xi '}} \end{aligned}$$

is obtained, corresponding to the far-field of a monopole charge.

The solution inside the ellipsoid is

87 $$\begin{aligned} \Phi _{\mathrm{in}}(\xi )=B\int \limits _{-c^2}^\xi \frac{d\xi '}{R_{\xi '}}. \end{aligned}$$

Using the asymptotic approximation $R_{\xi \rightarrow -c^2}\propto \sqrt{\xi +c^2}$ we obtain

88 $$\begin{aligned} \Phi _{\mathrm{in}}(\xi \rightarrow -c^2)\approx B\sqrt{\xi +c^2}. \end{aligned}$$

This solution satisfies the boundary condition $\lim _{\xi \rightarrow -c^2}\Phi (\xi )=0$. The constant *B* can be found from the boundary condition $\Phi (\xi =0)=V$, where V is the potential on the surface of the charged ellipsoid. Thus, $B=V/c$ and

89 $$\begin{aligned} \Phi _{\mathrm{in}}(\xi )=\frac{V}{c}\sqrt{\xi +c^2}. \end{aligned}$$

### Dipole moment of conducting ellipsoid induced by an external electric field in *z*-direction

Following Ref.^32^, let us consider the case when the external electric field is parallel to one of the major axes of the ellipsoid. For the external potential let us choose

90 $$\begin{aligned} \Phi _0=-E_0 z=-E_0 \sqrt{\frac{(\xi +c^2)(\eta +c^2)(\zeta +c^2)}{(a^2-c^2)(b^2-c^2)}} \end{aligned}$$

Let $\Phi _p$ be the potential caused by the ellipsoid, with the boundary condition $\Phi _p(\xi \rightarrow \infty )=0$. Requiring continuous boundary condition on the surface of the ellipsoid, we have

91 $$\begin{aligned} \Phi _{\mathrm{in}}(0,\eta ,\zeta )=\Phi _{0}(0,\eta ,\zeta )+\Phi _{p}(0,\eta ,\zeta ). \end{aligned}$$

We make the ansatz

92 $$\begin{aligned} \Phi _p(\xi ,\eta ,\zeta )& = {} F_p(\xi )\sqrt{(\eta +c^2)(\zeta +c^2)}, \end{aligned}$$

which after insertion into the Laplace equation yields

93 $$\begin{aligned} R_\xi \frac{d}{d\xi }\left[ R_\xi \frac{dF}{d\xi }\right] -\left( \frac{a^2+b^2}{4}+\frac{\xi }{2}\right) F(\xi )=0. \end{aligned}$$

Thus, one obtains for the field caused by the ellipsoid

94 $$\begin{aligned} \Phi _p(\xi ,\eta ,\zeta )=C_pF_p(\xi )\sqrt{(\eta +c^2)(\zeta +c^2)} \end{aligned}$$

with

95 $$\begin{aligned} F_p(\xi )=F_{\mathrm{in}}(\xi )\int \limits _\xi ^\infty \frac{d\xi '}{F_{\mathrm{in}}^2(\xi ')R_{\xi '}}, \end{aligned}$$

where

96 $$\begin{aligned} F_{\mathrm{in}}(\xi )=\sqrt{\xi +c^2}, \end{aligned}$$

the function we used in the case of the charged ellipsoid (see above). Thus, the field inside the ellipsoid is given by

97 $$\begin{aligned} \Phi _{\mathrm{in}}=C_{\mathrm{in}}F_{\mathrm{in}}(\xi )\sqrt{(\eta +c^2)(\zeta +c^2)}. \end{aligned}$$

Using the boundary condition shown in Eq. (91), one obtains the first equation

98 $$\begin{aligned} C_p\int \limits _0^\infty \frac{d\xi '}{(c^2+\xi ')R_{\xi '}}-C_{\mathrm{in}}=\frac{E_0}{\sqrt{(a^2-c^2)(b^2-c^2)}}, \end{aligned}$$

The boundary condition of the normal component of $\mathbf{D}$ at $\xi =0$, equivalent to

99 $$\begin{aligned} \varepsilon _{\mathrm{in}}\frac{\partial \Phi _{\mathrm{in}}}{\partial \xi }=\varepsilon _m\frac{\partial \Phi _0}{\partial \xi }+\varepsilon _m\frac{\partial \Phi _p}{\partial \xi }, \end{aligned}$$

yields the second equation

100 $$\begin{aligned} \varepsilon _mC_p\left[ \int \limits _0^\infty \frac{d\xi '}{(c^2+\xi ')R_{\xi '}}-\frac{2}{abc}\right] -\varepsilon _{\mathrm{in}}C_{\mathrm{in}} \nonumber \\ =\frac{\varepsilon _mE_0}{\sqrt{(a^2-c^2)(b^2-c^2)}}. \end{aligned}$$

Consequently, the potentials are

101 $$\begin{aligned} \Phi _{\mathrm{in}}& = {} \frac{\Phi _0}{1+\frac{L_3(\varepsilon _{\mathrm{in}}-\varepsilon _m)}{\varepsilon _m}}, \end{aligned}$$

102 $$\begin{aligned} \Phi _p& = {} \Phi _0\frac{\frac{abc}{2}\frac{\varepsilon _m-\varepsilon _{\mathrm{in}}}{\varepsilon _m}\int \limits _\xi ^\infty \frac{d\xi '}{(c^2+\xi ')R_{\xi '}}}{1+\frac{L_3(\varepsilon _{\mathrm{in}}-\varepsilon _m)}{\varepsilon _m}}, \end{aligned}$$

where

103 $$\begin{aligned} L_3=\frac{abc}{2}\int \limits _0^\infty \frac{d\xi '}{(c^2+\xi ')R_{\xi '}}. \end{aligned}$$

Far away from the ellipsoid for $\xi \approx r^2\rightarrow \infty$, one can use the approximation

104 $$\begin{aligned} \int \limits _\xi ^\infty \frac{d\xi '}{(c^2+\xi ')R_{\xi '}}\approx \int \limits _\xi ^\infty \frac{d\xi '}{{\xi '}^{5/2}}=\frac{2}{3}\xi ^{-3/2}, \end{aligned}$$

yielding the potential caused by the ellipsoid, i.e.

105 $$\begin{aligned} \Phi _p\approx \frac{E_0\cos \theta }{r^2}\frac{\frac{abc}{3}\frac{\varepsilon _{\mathrm{in}}-\varepsilon _m}{\varepsilon _m}}{1+\frac{L_3(\varepsilon _{\mathrm{in}}-\varepsilon _m)}{\varepsilon _m}}, \end{aligned}$$

from which we identify the dipole moment

106 $$\begin{aligned} \mathbf{p}=p\hat{\mathbf{z}}=4\pi \varepsilon _m abc\frac{\varepsilon _{\mathrm{in}}-\varepsilon _m}{3\varepsilon _m+3L_3(\varepsilon _{\mathrm{in}}-\varepsilon _m)}E_0\hat{\mathbf{z}}. \end{aligned}$$

This result determines the polarizability of the charged ellipsoid, i.e.

107 $$\begin{aligned} \alpha _3=4\pi \varepsilon _m abc\frac{\varepsilon _{\mathrm{in}}-\varepsilon _m}{3\varepsilon _m+3L_3(\varepsilon _{\mathrm{in}}-\varepsilon _m)} \end{aligned}$$

If the external electric field is applied along the other major axes of the ellipsoid, *x* or *y*, the polarizabilities are

108 $$\begin{aligned} \alpha _1& = {} 4\pi \varepsilon _m abc\frac{\varepsilon _{\mathrm{in}}-\varepsilon _m}{3\varepsilon _m+3L_1(\varepsilon _{\mathrm{in}}-\varepsilon _m)}, \end{aligned}$$

109 $$\begin{aligned} \alpha _2& = {} 4\pi \varepsilon _m abc\frac{\varepsilon _{\mathrm{in}}-\varepsilon _m}{3\varepsilon _m+3L_2(\varepsilon _{\mathrm{in}}-\varepsilon _m)}, \end{aligned}$$

respectively, where

110 $$\begin{aligned} L_1& = {} \frac{abc}{2}\int \limits _0^\infty \frac{d\xi '}{(a^2+\xi ')R_{\xi '}}, \end{aligned}$$

111 $$\begin{aligned} L_2& = {} \frac{abc}{2}\int \limits _0^\infty \frac{d\xi '}{(b^2+\xi ')R_{\xi '}}. \end{aligned}$$

For oblate spheroids ($a=b$), $L_1=L_2$,

112 $$\begin{aligned} L_1& = {} \frac{g(e_o)}{2e_o^2}\left[ \frac{\pi }{2}-\arctan g(e_o)\right] -\frac{g^2(e_o)}{2}, \nonumber \\ g(e_o)& = {} \sqrt{\frac{1-e_o^2}{e_o^2}},\; e_o^2=1-\frac{c^2}{a^2}, \end{aligned}$$

where $e_o$ is the eccentricity of the oblate spheroid. The limiting cases of an infinitesimally thin disk and a sphere are obtained for $e_o=1$ and $e_o=0$, respectively.

The geometrical factors $L_i$ are related to the depolarization factors ${\hat{L}}_i$ by

113 $$\begin{aligned} E_{\mathrm{in}x}& = {} E_{0x}-{\hat{L}}_1P_{\mathrm{in}x},\end{aligned}$$

114 $$\begin{aligned} E_{\mathrm{in}y}& = {} E_{0y}-{\hat{L}}_2P_{\mathrm{in}y},\end{aligned}$$

115 $$\begin{aligned} E_{\mathrm{in}z}& = {} E_{0z}-{\hat{L}}_3P_{\mathrm{in}z}, \end{aligned}$$

with

116 $$\begin{aligned} {\hat{L}}_i=\frac{\varepsilon _{\mathrm{in}}-\varepsilon _m}{\varepsilon _{\mathrm{in}}-\varepsilon _0}\frac{L_i}{\varepsilon _m}. \end{aligned}$$

### Dipole moment of conducting ellipsoid induced by an external electric field in *x*-direction

In analogy to Ref.^32^, let us consider the case when the external electric field is parallel to one of the major axes of the ellipsoid, in this case along the *x*-axis. For the external potential let us choose

117 $$\begin{aligned} \Phi _0=-E_0 x=-E_0 \sqrt{\frac{(\xi +a^2)(\eta +a^2)(\zeta +a^2)}{(b^2-a^2)(c^2-a^2)}}. \end{aligned}$$

Let $\Phi _p$ be the potential caused by the ellipsoid, with the boundary condition $\Phi _p(\xi \rightarrow \infty )=0$. Requiring continuous boundary condition on the surface of the ellipsoid, we have

118 $$\begin{aligned} \Phi _{\mathrm{in}}(0,\eta ,\zeta )=\Phi _0(0,\eta ,\zeta )+\Phi _p(0,\eta ,\zeta ). \end{aligned}$$

Thus, one obtains for the field caused by the ellipsoid

119 $$\begin{aligned} \Phi _p(\xi ,\eta ,\zeta )=C_pF_p(\xi )\sqrt{(\eta +a^2)(\zeta +a^2)} \end{aligned}$$

with

120 $$\begin{aligned} F_p(\xi )=F_{\mathrm{in}}(\xi )\int \limits _\xi ^\infty \frac{d\xi '}{F_{\mathrm{in}}^2(\xi ')R_{\xi '}}, \end{aligned}$$

where

121 $$\begin{aligned} F_{\mathrm{in}}(\xi )=\sqrt{\xi +a^2}, \end{aligned}$$

the function we used in the case of the charged ellipsoid (see above). Thus, the field inside the ellipsoid is given by

122 $$\begin{aligned} \Phi _{\mathrm{in}}=C_{\mathrm{in}}F_{\mathrm{in}}(\xi )\sqrt{(\eta +a^2)(\zeta +a^2)}. \end{aligned}$$

Using the boundary condition shown in Eq. (118), one obtains the first equation

123 $$\begin{aligned} C_p\int \limits _0^\infty \frac{d\xi '}{(a^2+\xi ')R_{\xi '}}-C_{\mathrm{in}}=\frac{E_0}{\sqrt{(b^2-a^2)(c^2-a^2)}}, \end{aligned}$$

The boundary condition of the normal component of $\mathbf{D}$ at $\xi =0$, equivalent to

124 $$\begin{aligned} \varepsilon _{\mathrm{in}}\frac{\partial \Phi _{\mathrm{in}}}{\partial \xi }=\varepsilon _m\frac{\partial \Phi _0}{\partial \xi }+\varepsilon _m\frac{\partial \Phi _p}{\partial \xi }, \end{aligned}$$

yields the second equation

125 $$\begin{aligned} \varepsilon _mC_p\left[ \int \limits _0^\infty \frac{d\xi '}{(a^2+\xi ')R_{\xi '}}-\frac{2}{abc}\right] -\varepsilon _{\mathrm{in}}C_{\mathrm{in}} \nonumber \\ =\frac{\varepsilon _mE_0}{\sqrt{(b^2-a^2)(c^2-a^2)}}. \end{aligned}$$

Consequently, the potentials are

126 $$\begin{aligned} \Phi _{\mathrm{in}}& = {} \frac{\Phi _0}{1+\frac{L_1(\varepsilon _{\mathrm{in}}-\varepsilon _m)}{\varepsilon _m}}, \end{aligned}$$

127 $$\begin{aligned} \Phi _p& = {} \Phi _0\frac{\frac{abc}{2}\frac{\varepsilon _m-\varepsilon _{\mathrm{in}}}{\varepsilon _m}\int \limits _\xi ^\infty \frac{d\xi '}{(a^2+\xi ')R_{\xi '}}}{1+\frac{L_1(\varepsilon _{\mathrm{in}}-\varepsilon _m)}{\varepsilon _m}}, \end{aligned}$$

where

128 $$\begin{aligned} L_1=\frac{abc}{2}\int \limits _0^\infty \frac{d\xi '}{(a^2+\xi ')R_{\xi '}}. \end{aligned}$$

Far away from the ellipsoid for $\xi \approx r^2\rightarrow \infty$, one can use the approximation

129 $$\begin{aligned} \int \limits _\xi ^\infty \frac{d\xi '}{(a^2+\xi ')R_{\xi '}}\approx \int \limits _\xi ^\infty \frac{d\xi '}{{\xi '}^{5/2}}=\frac{2}{3}\xi ^{-3/2}, \end{aligned}$$

yielding the potential caused by the ellipsoid, i.e.

130 $$\begin{aligned} \Phi _p\approx \frac{E_0\cos \theta }{r^2}\frac{\frac{abc}{3}\frac{\varepsilon _{\mathrm{in}}-\varepsilon _m}{\varepsilon _m}}{1+\frac{L_1(\varepsilon _{\mathrm{in}}-\varepsilon _m)}{\varepsilon _m}}, \end{aligned}$$

from which we identify the dipole moment

131 $$\begin{aligned} \mathbf{p}=p\hat{\mathbf{x}}=4\pi \varepsilon _m abc\frac{\varepsilon _{\mathrm{in}}-\varepsilon _m}{3\varepsilon _m+3L_1(\varepsilon _{\mathrm{in}}-\varepsilon _m)}E_0\hat{\mathbf{x}}. \end{aligned}$$

This result determines the polarizability of the charged ellipsoid, i.e.

132 $$\begin{aligned} \alpha _1=4\pi \varepsilon _m abc\frac{\varepsilon _{\mathrm{in}}-\varepsilon _m}{3\varepsilon _m+3L_1(\varepsilon _{\mathrm{in}}-\varepsilon _m)} \end{aligned}$$

### Dipole moment of conducting single-sheet hyperboloid with a small elliptical wormhole induced by an external electric field

Let us consider a conducting single-sheet hyperboloid with a small elliptical wormhole, as shown in Fig. 11. Contrary to the case of an uncharged ellipsoid, where the solutions when applying the external electric field in *x*, *y*, or *z* direction are similar, the solutions in the case of an uncharged hyperboloid depend strongly on the axis in which the external field $\mathbf{E}_0$ points. While the solutions for $\mathbf{E}_0=E_0\hat{\mathbf{x}}$ and $\mathbf{E}_0=E_0\hat{\mathbf{y}}$ are similar, the solution for $\mathbf{E}_0=E_0\hat{\mathbf{z}}$ is completely different. The reason for this fundamental difference is that the ellipsoid resembles a sphere from far away. However, a single-sheet hyperboloid has elliptical cylindrical symmetry.

Here, let us first calculate the electrostatic potential $\Phi (\xi ,\eta ,\zeta )$ of a conducting single-sheet hyperboloid with an elliptical hole, which can be represented by a limiting hyperboloid from a family of hyperboloids described by the implicit equation

133 $$\begin{aligned} \frac{x^2}{a^2+u}+\frac{y^2}{b^2+u}+\frac{z^2}{c^2+u}=1 \end{aligned}$$

for $a>b>c$. The cubic roots $\xi$, $\eta$, and $\zeta$ are all real in the ranges

134 $$\begin{aligned} -a^2\le \zeta \le -b^2,\; -b^2\le \eta \le -c^2,\; -c^2\le \xi <\infty , \end{aligned}$$

which are the ellipsoidal coordinates of a point (*x*, *y*, *z*). The lmiting hyperboloid is a single planar sheet with an elliptical hole, i.e. it belongs to the family of solutions $\eta$ in the limit $\eta \rightarrow -c^2$. Therefore, let us choose this limiting case as our origin in ellipsoidal coordinates with $c=0$. Then Eq. (133) becomes

135 $$\begin{aligned} \frac{x^2}{a^2+u}+\frac{y^2}{b^2+u}+\frac{z^2}{u}=1 \end{aligned}$$

for $a>b>c=0$. The cubic roots $\xi$, $\eta$, and $\zeta$ are all real in the ranges

136 $$\begin{aligned} -a^2\le \zeta \le -b^2,\; -b^2\le \eta \le 0,\; 0\le \xi <\infty , \end{aligned}$$

The surface of the conducting hyperboloid is defined by $-b^2\le \eta =\eta _1<0$.

Let us consider the case $\mathbf{E}_0=E_0\hat{\mathbf{x}}$, which in the limit when the hyperboloid becomes a flat plane is the most relevant one. Therefore

137 $$\begin{aligned} \Psi _0=-E_0 x=\mp E_0 \sqrt{\frac{(\xi +a^2)(\eta +a^2)(\zeta +a^2)}{(b^2-a^2)(-a^2)}} \end{aligned}$$

in the lower-half plane, where the negative sign corresponds to positive *x* values and the positive sign to negative *x* values. Since the equipotential surfaces are determined by $\eta$, let $\Psi _p$ be the potential caused by the hyperboloid, with the boundary condition $\Psi _{\mathrm{in}}(\eta =0)=0$. Requiring continuous boundary condition on the surface of the hyperboloid, we have

138 $$\begin{aligned} \Psi _{\mathrm{in}}(\xi ,\eta _1,\zeta )& = {} \Psi _0(\xi ,\eta _1,\zeta )+\Psi _p(\xi ,\eta _1,\zeta ) , \end{aligned}$$

139 $$\begin{aligned} \varepsilon _{\mathrm{in}}\left. \frac{\partial \Psi _{\mathrm{in}}}{\partial \eta }\right| _{\eta _1}& = {} \varepsilon _m\left. \frac{\partial \Psi _0}{\partial \eta }\right| _{\eta _1}+\varepsilon _m\left. \frac{\partial \Psi _p}{\partial \eta }\right| _{\eta _1}, \end{aligned}$$

where in the second equation the normal component of $\mathbf{D}$ at $\eta =\eta _1$ must be continuous. Then we make the ansatz for the electrostatic potential inside the hyperboloid,

140 $$\begin{aligned} \Psi _{\mathrm{in}}(\xi ,\eta ,\zeta )=-C_{\mathrm{in}}E_0x, \end{aligned}$$

where $C_{\mathrm{in}}$ is a constant. This ansatz satisfies the boundary condition $\Psi _{\mathrm{in}}(\xi =0,\eta \rightarrow 0,\zeta )=0$. For the outside polarization field we choose

141 $$\begin{aligned} \Psi _p(\xi ,\eta ,\zeta ) = -C_pE_0xF_1(\xi )K_1(\eta ) \end{aligned}$$

where $C_p$ is a constant, and we defined

142 $$\begin{aligned} F_1(\xi )& = {} \int \limits _{\xi }^{\infty } \frac{ad\xi '}{2{\xi '}^{1/2}(\xi '+a^2)}- \int \limits _{\xi }^{\infty } \frac{ad\xi '}{2(\xi '+a^2)^{3/2}} \nonumber \\& = {} \arctan \left( \frac{a}{\sqrt{\xi }}\right) -\frac{a}{\sqrt{\xi +a^2}}. \end{aligned}$$

Note that $\lim _{\xi \rightarrow 0_+}\arctan \left( \frac{a}{\sqrt{\xi }}\right) =\pi /2$, whereas $\lim _{\xi \rightarrow 0_-}\arctan \left( \frac{a}{\sqrt{\xi }}\right) =-\pi /2$. Therefore, in order to avoid discontinuity at $\xi =0$, we must have $\arctan \left( \frac{a}{-\sqrt{\xi }}\right) =\pi -\arctan \left( \frac{a}{\sqrt{\xi }}\right)$.

143 $$\begin{aligned} K_1(\eta )=\int \limits _{\eta }^\infty \frac{d\eta '}{(\eta '+a^2)R_{\eta '}}, \end{aligned}$$

where $R_\eta = \sqrt{(\eta +a^2)(\eta +b^2)(-\eta )}$. The boundary conditions at $z\rightarrow \pm \infty$ are satisfied:

144 $$\begin{aligned} F_1(\xi ) =\left\{ \begin{array}{cc} 0 &{} \text{ for } z\rightarrow +\infty \\ \pi &{} \text{ for } z\rightarrow -\infty \end{array} \right. . \end{aligned}$$

At large distances $r=\sqrt{x^2+y^2+z^2}$ from the wormhole we have $\xi \approx r^2$. Then the far-field potential in the upper half-space, which is given by the pure polarization field, is

145 $$\begin{aligned} \Psi _p(\xi ,\eta ,\zeta )\approx & {} -C_pE_0x K_1(\eta \approx -b^2)\frac{1}{3}\left( \frac{a}{\sqrt{\xi }}\right) ^3 \nonumber \\\approx & {} -C_pE_0 K_1(\eta \approx -b^2) \frac{a^3}{3}\frac{x}{r^3}. \end{aligned}$$

The polarization far-field has the form of a dipole field at large distances *r* from the wormhole.

In order to determine the polarizability of the wormhole, let us find the solution at $\xi =0$, corresponding to the plane that passes through the center of the wormhole. For $\xi =0$, the unit vectors $\hat{\mathbf{x}}$ and $\hat{{\varvec{\eta }}}$ are parallel. In this near-field limit, the polarization potential has the form

146 $$\begin{aligned} \Psi _p(\xi ,\eta ,\zeta ) = -{\tilde{C}}_pE_0x K_1(\eta ), \end{aligned}$$

where $${\tilde{C}}_p=C_p\left( \pi /2-1\right)$$.

Using the boundary conditions shown in Eq. (139), we obtain the first equation

147 $$\begin{aligned} {\tilde{C}}_pK_1(\eta _1)-C_{\mathrm{in}} = 1, \end{aligned}$$

and the second equation

148 $$\begin{aligned}&\varepsilon _m{\tilde{C}}_p\left[ K_1(\eta _1)\left. \frac{\partial x}{\partial \eta }\right| _{\eta _1}+K_1'(\eta _1)\left. x\right| _{\eta _1}\right] \nonumber \\&-\varepsilon _{\mathrm{in}}C_{\mathrm{in}}\left. \frac{\partial x}{\partial \eta }\right| _{\eta _1} = \varepsilon _m\left. \frac{\partial x}{\partial \eta }\right| _{\eta _1}. \end{aligned}$$

Using the derivatives

149 $$\begin{aligned} \left. \frac{\partial x}{\partial \eta }\right| _{\xi =0,\eta _1}& = {} \frac{a}{2}\sqrt{\frac{(\zeta +a^2)}{(\eta _1+a^2)(a^2-b^2)(a^2-c^2)}}, \end{aligned}$$

150 $$\begin{aligned} K_1'(\eta _1)& = {} \frac{1}{(\eta _1+a^2)R_{\eta _1}} \end{aligned}$$

we can rewrite the second equation as

151 $$\begin{aligned}&\varepsilon _m{\tilde{C}}_p\left[ \frac{K_1(\eta _1)}{\eta _1+a^2}+K_1'(\eta _1)\right] \nonumber \\&-\varepsilon _{\mathrm{in}}C_{\mathrm{in}}\frac{1}{\eta _1+a^2} = \varepsilon _m\frac{1}{\eta _1+a^2}, \end{aligned}$$

which is equivalent to

152 $$\begin{aligned}&\varepsilon _m{\tilde{C}}_p\left[ K_1(\eta _1)+\frac{1}{R_{\eta _1}}\right] \nonumber \\&-\varepsilon _{\mathrm{in}}C_{\mathrm{in}} = \varepsilon _m . \end{aligned}$$

Thus, the potentials are

153 $$\begin{aligned} \Psi _{\mathrm{in}}& = {} \frac{\Psi _0}{1+\frac{L_1(\varepsilon _{\mathrm{in}}-\varepsilon _m)}{\varepsilon _m}}, \end{aligned}$$

154 $$\begin{aligned} \Psi _p& = {} \Psi _0\frac{R_{\eta _1}\frac{\varepsilon _m-\varepsilon _{\mathrm{in}}}{\varepsilon _m}F_1(\xi )K_1(\eta )(\pi /2-1)}{1+\frac{L_1(\varepsilon _{\mathrm{in}}-\varepsilon _m)}{\varepsilon _m}}. \end{aligned}$$

Then the far-field potential in the upper half-space, which is given by the pure polarization field, is

155 $$\begin{aligned} \Psi _p\approx & {} -E_0\frac{R_{\eta _1}\frac{\varepsilon _m-\varepsilon _{\mathrm{in}}}{\varepsilon _m} K_1(\eta \approx -b^2)(\pi /2-1)}{1+\frac{L_1(\varepsilon _{\mathrm{in}}-\varepsilon _m)}{\varepsilon _m}} \frac{a^3}{3}\frac{x}{r^3} \nonumber \\\approx & {} -E_0\frac{ab\sqrt{-\eta _1}\frac{\varepsilon _m-\varepsilon _{\mathrm{in}}}{\varepsilon _m} \frac{2\pi }{a^3}(\pi /2-1)}{1+\frac{L_1(\varepsilon _{\mathrm{in}}-\varepsilon _m)}{\varepsilon _m}} \frac{a^3}{3}\frac{x}{r^3} \nonumber \\& = {} -E_0\frac{ab\sqrt{-\eta _1}\frac{\varepsilon _m-\varepsilon _{\mathrm{in}}}{\varepsilon _m} \pi (\pi /2-1)}{1+\frac{L_1(\varepsilon _{\mathrm{in}}-\varepsilon _m)}{\varepsilon _m}} \frac{2x}{3r^3}, \end{aligned}$$

where we assumed that $a\approx b$. The polarization far-field has the form of a dipole field at large distances *r* from the wormhole. If the external electric field is applied in *y*-direction, we obtain the potentials

156 $$\begin{aligned} \Psi _{\mathrm{in}}& = {} \frac{\Psi _0}{1+\frac{L_2(\varepsilon _{\mathrm{in}}-\varepsilon _m)}{\varepsilon _m}}, \end{aligned}$$

157 $$\begin{aligned} \Psi _p& = {} \Psi _0\frac{R_{\eta _1}\frac{\varepsilon _m-\varepsilon _{\mathrm{in}}}{\varepsilon _m}F_2(\xi )K_2(\eta )(\pi -1)}{1+\frac{L_2(\varepsilon _{\mathrm{in}}-\varepsilon _m)}{\varepsilon _m}}, \end{aligned}$$

with

158 $$\begin{aligned} F_2(\xi )& = {} \int \limits _{\xi }^{\infty } \frac{bd\xi '}{2{\xi '}^{1/2}(\xi '+b^2)}- \int \limits _{\xi }^{\infty } \frac{bd\xi '}{2(\xi '+b^2)^{3/2}}, \end{aligned}$$

159 $$\begin{aligned} K_2(\eta )& = {} \int \limits _{\eta }^\infty \frac{d\eta '}{(\eta '+b^2)R_{\eta '}}. \end{aligned}$$

We defined the geometrical factors

160 $$\begin{aligned} L_1& = {} R_{\eta _1}K_1(\eta _1)\approx ab\sqrt{-\eta _1}\int \limits _{\eta _1}^\infty \frac{d\eta '}{(\eta '+a^2)R_{\eta '}}, \end{aligned}$$

161 $$\begin{aligned} L_2& = {} R_{\eta _1}K_2(\eta _1)\approx ab\sqrt{-\eta _1}\int \limits _{\eta _1}^\infty \frac{d\eta '}{(\eta '+b^2)R_{\eta '}}, \end{aligned}$$

which are related to the depolarization factors by

162 $$\begin{aligned} {\tilde{L}}_i = \frac{\varepsilon _{\mathrm{in}}-\varepsilon _m}{\varepsilon _{\mathrm{in}}-\varepsilon _0}\frac{L_i}{\varepsilon _m}. \end{aligned}$$

This result determines the polarizability of the uncharged hyperboloid observable in the far-field, i.e.

163 $$\begin{aligned} \alpha _1= \frac{2ab\sqrt{-\eta _1}\pi (\pi /2-1)}{3}\frac{\varepsilon _{\mathrm{in}}-\varepsilon _m}{\varepsilon _m+L_1(\varepsilon _{\mathrm{in}}-\varepsilon _m)}. \end{aligned}$$

Similarly, we obtain the polarizability in *y*-direction, i.e.

164 $$\begin{aligned} \alpha _2 = \frac{2ab\sqrt{-\eta _1}\pi (\pi /2-1)}{3}\frac{\varepsilon _{\mathrm{in}}-\varepsilon _m}{\varepsilon _m+L_2(\varepsilon _{\mathrm{in}}-\varepsilon _m)}. \end{aligned}$$

Comparing to the polarizabilities of ellipsoids^32^, the polarizabilities of hyperboloids are proportional to $ab\sqrt{-\eta _1}$, which corresponds to the volume of the ellipsoid *abc*.

In the case of circular wormholes, we have $a=b$, and therefore $\alpha _1=\alpha _2=\alpha _{||}$, with $L_1=L_2=L_{||}$.

### Dispersion relations

In our proposed mid-IR light source the effective combination of silicon nitride ($\hbox {Si}_3\,\hbox {N}_4$) and hexagonal boron nitride (h-BN) behaves as an environment with polar phonons. Both materials are polar with ions of different valence, which leads to the Frohlich interaction between electrons and optical phonons^33^. Fig. 12 shows that the interaction between the electrons in graphene and the polar substrate/graphene phonons modifies substantially the dispersion relations for the surface plasmon polaritons in graphene. The RPA dielectric function of graphene is given by^11,12^

165 $$\begin{aligned} {\mathbf{{\varepsilon }}^{RPA}}(\mathbf{{q}},\omega )& = {} {\mathbf{{\varepsilon }}_m} - {v_c}(\mathbf{{q}}){\chi ^0}(\mathbf{{q}},\omega ) \nonumber \\&\quad - {\mathbf{{\varepsilon }}_m}\sum \limits _l {{v_{sph,l}}(\mathbf{{q}},\omega )} {\chi ^0}(\mathbf{{q}},\omega ) \nonumber \\&- {\mathbf{{\varepsilon }}_m}{v_{oph}}(\mathbf{{q}},\omega )\chi _{j,j}^0(\mathbf{{q}},\omega ). \end{aligned}$$

The second term is due to the effective Coulomb interaction, and ${v_c}(\mathbf{{q}}) = {{{e^2}}/{2q}}{\varepsilon _0}$ is the 2D Coulomb interaction. The effective electron-electron interaction mediated by the substrate optical phonons,

166 $$\begin{aligned} {v_{sph,l}}(\mathbf{{q}},\omega ) = {\left| {{M_{sph}}} \right| ^2}G_l^0(\omega ), \end{aligned}$$

gives rise to the third term, where $|M_{sph} |^2$ is the scattering and $G_l^0$ is the free phonon Green function. The effective electron-electron interaction due to the optical phonons in graphene,

167 $$\begin{aligned} {v_{oph}}(\mathbf{{q}},\omega ) = {\left| {{M_{oph}}} \right| ^2}{G^0}(\omega ), \end{aligned}$$

gives rise to the last term of Eq. (165). $|M_oph |^2$ is the scattering matrix element, and ${G^o}(\omega )$ is the free phonon Green function. $\chi _{j,j}^0(\mathbf{{q}},\omega )$ is the current-current correlation function in Eq. (165).

The relaxation time $\tau$ of the momentum consists of the electron-impurity, electron-phonon, and the electron-edge scattering, $\tau ^{-1}=\tau _{DC}^{-1}+\tau _{edge}^{-1}+\tau _{e-p}^{-1}$, which determines the plasmon lifetime and the absorption spectrum bandwidth. It can be evaluated via the measured DC mobility $\mu$ of the graphene sample through ${\tau _{DC}} = {{\mu \hbar \sqrt{\pi \rho } }/{e{v_F}}}$, where $v_F=10^6$ m/s is the Fermi velocity, and the charge carrier density is given by ${\tau _{DC}} = {{\mu \hbar \sqrt{\pi \rho } }/{e{v_F}}}$. The edge scattering time is ${\tau _{edge}} \approx {\left( {{{1 \times {{10}^6}({m/s})}/{w - {w_0}}}} \right) ^{ - 1}}$, where *w* is the edge-to-edge distance between the holes, and $w_0=7$ nm is the adjustment parameter. The electron-phonon scattering time is ${\tau _{e - ph}} = {\hbar /{2{\mathop {\mathrm{Im}}\nolimits } ({\sum _{e - ph}})}}$. The imaginary part of the electron-phonon self-energy reads

168 $$\begin{aligned} {\mathop {\mathrm{Im}}\nolimits } \left( {\sum _{e - ph}}\right) = \gamma \left| {\hbar \omega - {\mathop {\mathrm{sgn}}} \left( {\hbar \omega - {E_F}} \right) \hbar {\omega _{oph}}} \right| , \end{aligned}$$

where $\gamma = 18.3 \times {10^{ - 3}}$ is the electron-phonon coupling coefficient. The optical phonon energy of graphene is given by $\hbar {\omega _{oph}} \approx 0.2$ eV.

The loss function *Z* describes the interaction of the SPPs and the substrate/graphene phonons. In RPA we have

169 $$\begin{aligned} Z \propto - {\mathop {\mathrm{Im}}\nolimits } \left( {\frac{1}{{{\varepsilon ^{RPA}}}}} \right) . \end{aligned}$$

Figure 12a shows the loss function for graphene with carrier mobility $\mu =3000$ $\hbox {cm}^2$/V$\cdot$s and a Fermi energy of $E_F=1.0$ eV. In order to take advantage of the enhancement of the electromagnetic field at the position of the graphene sheet, the thickness of the optical cavity must be $\lambda /4n$, where *n* is the refractive index of the cavity material^11^. The LSP resonance frequencies $\omega _{LSP4}$, $\omega _{LSP7}$, and $\omega _{LSP10}$ mark the frequencies around the resonance wavelengths of 4 $\upmu$m, 7 $\upmu$m. and 10 $\upmu$m. The resonance frequencies of the polar phonons are denoted by $\omega _{BN}$ for h-BN and by $\omega _{SN}$ for $\hbox {Si}_3\,\hbox {N}_4$.

### Integral of dyadic Green function elements over spherical angle

For the calculation of the spectral radiance we need to integrate the elements of the dyadic Green function over the spherical angle. We can split the total dyadic Green function into a free space term $\overleftrightarrow {\mathbf{G}}_0(\mathbf{r},\mathbf{r}';\omega )$ and a term $\overleftrightarrow {\mathbf{G}}_{SPP}(\mathbf{r},\mathbf{r}';\omega )$ that creates surface plasmon polaritons inside graphene. Since the absorbance of the pristine graphene sheet is only 2.3%, we can safely neglect $\overleftrightarrow {\mathbf{G}}_{SPP}(\mathbf{r},\mathbf{r}';\omega )$. Our goal is to calculate the gray-body emission of the EM radiation from the LSP around the holes in graphene into free space. Therefore, we need to evaluate

170 $$\begin{aligned} I_{GB}^\infty (\omega )=\lim _{r\rightarrow \infty }\int r^2\sin \theta d\theta d\varphi I_{GB}(r,\omega ), \end{aligned}$$

where can use the approximation

171 $$\begin{aligned} I_{GB}(r,\omega )=I_0(r,\omega )-I_{SPP}(r,\omega )\approx I_0(r,\omega ). \end{aligned}$$

In Cartesian coordinates, we can write down the dyadic Green function as^19^

172 $$\begin{aligned} \overleftrightarrow {\mathbf{G}}_0(\mathbf{r};\omega )& = {} \frac{e^{ikr}}{4\pi r}\left[ \left( 1+\frac{i}{kr}-\frac{1}{k^2r^2}\right) \overleftrightarrow {\mathbb {1}}\right. \nonumber \\&\quad +\left. \left( \frac{3}{k^2r^2}-\frac{3i}{kr}-1\right) \hat{\mathbf{r}}\hat{\mathbf{r}}\right] . \end{aligned}$$

Since we are interested only in the far field, we consider only the far-field component of the dyadic Green function, which is

173 $$\begin{aligned} \overleftrightarrow {\mathbf{G}}_{FF}(\mathbf{r};\omega ) = \frac{e^{ikr}}{4\pi r}\left[ \overleftrightarrow {\mathbb {1}}- \hat{\mathbf{r}}\hat{\mathbf{r}}\right] , \end{aligned}$$

which possesses only angular (transverse) components but no radial (longitudinal) components. Then the necessary components are

174 $$\begin{aligned} G_{xx}(\mathbf{r};\omega )& = {} \frac{e^{ikr}}{4\pi r}\left[ 1-\sin ^2\theta \cos ^2\varphi \right] , \nonumber \\ G_{yx}(\mathbf{r};\omega )& = {} \frac{e^{ikr}}{4\pi r}\left[ 1-\sin ^2\theta \cos \varphi \sin \varphi \right] \nonumber \\ G_{zx}(\mathbf{r};\omega )& = {} \frac{e^{ikr}}{4\pi r}\left[ 1-\sin \theta \cos \theta \cos \varphi \right] , \nonumber \\ G_{xy}(\mathbf{r};\omega )& = {} \frac{e^{ikr}}{4\pi r}\left[ 1-\sin ^2\theta \cos \varphi \sin \varphi \right] , \nonumber \\ G_{yy}(\mathbf{r};\omega )& = {} \frac{e^{ikr}}{4\pi r}\left[ 1-\sin ^2\theta \sin ^2\varphi \right] , \nonumber \\ G_{zy}(\mathbf{r};\omega )& = {} \frac{e^{ikr}}{4\pi r}\left[ 1-\sin \theta \cos \theta \sin \varphi \right] , \end{aligned}$$

The corresponding integrals are

175 $$\begin{aligned} \int r^2\sin \theta d\theta d\varphi \left| G_{xx}(\mathbf{r};\omega )\right| ^2& = {} \frac{2}{15\pi }, \nonumber \\ \int r^2\sin \theta d\theta d\varphi \left| G_{yx}(\mathbf{r};\omega )\right| ^2& = {} \frac{4}{15\pi }, \nonumber \\ \int r^2\sin \theta d\theta d\varphi \left| G_{zx}(\mathbf{r};\omega )\right| ^2& = {} \frac{4}{15\pi }, \nonumber \\ \int r^2\sin \theta d\theta d\varphi \left| G_{xy}(\mathbf{r};\omega )\right| ^2& = {} \frac{4}{15\pi }, \nonumber \\ \int r^2\sin \theta d\theta d\varphi \left| G_{yy}(\mathbf{r};\omega )\right| ^2& = {} \frac{2}{15\pi }, \nonumber \\ \int r^2\sin \theta d\theta d\varphi \left| G_{zy}(\mathbf{r};\omega )\right| ^2& = {} \frac{4}{15\pi }. \end{aligned}$$

### Doping of graphene due to $\hbox {Si}_3\,\hbox {N}_4$

The Silicon nitride, $\hbox {Si}_3\,\hbox {N}_4$, dielectric layer causes an effective n-type doping in graphene sheet^34,35^. The shift in Fermi energy is given by

176 $$\begin{aligned} {E_F} = \hbar {v_F}\sqrt{\pi n}, \end{aligned}$$

where $v_F$ is the Fermi velocity (${v_F} \approx {10^6}m/s$ for graphene), $\hbar$ is Planck’s constant, and *n* is the carrier density. The carrier density *n* depends on the gate voltage and capacitance, i.e.

177 $$\begin{aligned} n=C\Delta V /e, \end{aligned}$$

where $\Delta V = \left( {{V_G} - {V_{CNP}}} \right)$ is the gate voltage relative to charge neutrality point, *e* is electric charge, and *C* is the capacitance of dielelectric layer, given by $C = \frac{{{\varepsilon _r}{\varepsilon _0}}}{d}$, ${\varepsilon _r}$ is the relative permittivity, $\mathrm{{\;}}{\varepsilon _0}$ is the permittivity of free space, and *d* is the thickness of dielectric layer.

The gate capacitance for a 50 nm thick $\hbox {Si}_3\,\hbox {N}_4$ layer in the infrared region is ${V_G} = 4.5\; \times {10^{ - 8}}F/c{m^2}$. From Eq. (176) we conclude that the Fermi energy $E_F=1$ eV corresponds to a gate voltage relative to the CNP of $\Delta V = \left( {{V_G} - {V_{CNP}}} \right) =6.9$ V.

Wang et al.^34^ observed that a $\hbox {Si}_3\,\hbox {N}_4$ film with a thickness of 50 nm shifts the CNP in a graphene sheet to -20 V, which shows that graphene is n-doped at zero gate voltage and the Fermi energy is $E_F=1.74$ eV. The Fermi energy can be tuned by applying a gate voltage to a desired value. In our work, we have used a Fermi energy of $E_F=1$ eV, which corresponds to $\Delta V=6.59$ V, i.e. for the CNP at − 20 V, $V_G= -13.41$ V results in a Fermi energy of $E_F=1$ eV. From Eqs. (176) and (177), the carrier density required to achieve a Fermi energy of $E_F=1$ eV is $n=1.94 \times {10^{12}}$ $\hbox {cm}^{ - 2}$, which corresponds to an electric field of ${E_{1.0 \mathrm {eV}}} = \frac{{en}}{{{\varepsilon _r}{\varepsilon _0}}} = 1.38 \times {10^6}$ $\hbox {Vcm}^{-1}$, which is in the safe zone compared to the reported breakdown field of the order of $10^7$ $\hbox {Vcm}^{-1}$ ^36^.
